# Inhibition of furin in CAR macrophages directs them toward a proinflammatory phenotype and enhances their antitumor activities

**DOI:** 10.1038/s41419-024-07267-4

**Published:** 2024-12-04

**Authors:** Lydia Ziane-Chaouche, Antonella Raffo-Romero, Nawale Hajjaji, Firas Kobeissy, Donna Pinheiro, Soulaimane Aboulouard, Adeline Cozzani, Suman Mitra, Isabelle Fournier, Dasa Cizkova, Michel Salzet, Marie Duhamel

**Affiliations:** 1 Université Lille, Inserm, CHU Lille, U1192, Laboratoire Protéomique, Réponse Inflammatoire Et Spectrométrie de Masse (PRISM), Villeneuve d’Ascq, France; 2 Equipe Labellisée Ligue Contre le Cancer, Villeneuve d’Ascq, France; 3 Breast Cancer Unit, Oscar Lambret Center, Lille, France; 4 Department of Biochemistry and Molecular Genetics, Faculty of Medicine, American University of Beirut, Beirut, Lebanon; 5 Inserm UMR1277, CNRS UMR9020-CANTHER, Université de Lille, Lille University Hospital, Lille, France; 6 Institute of Neuroimmunology, Slovak Academy of Sciences, Dúbravská cesta 9, Bratislava, Slovakia; 7 Centre for Experimental and Clinical Regenerative Medicine, Clinic of Small Animals, University of Veterinary Medicine and Pharmacy in Kosice, Kosice, Slovakia

**Keywords:** Tumour immunology, Immunotherapy

## Abstract

Chimeric antigen receptor (CAR)-T-cell therapy has revolutionized cellular immunotherapy, demonstrating remarkable efficacy in hematological cancers. However, its application in solid tumors faces significant challenges, including limited T-cell infiltration and tumor-induced immunosuppression. Given the prominent role of macrophages in the tumor microenvironment, their phenotypic plasticity and inherent antitumor properties, such as phagocytosis, offer a promising avenue for therapeutic intervention. This study focuses on the development of a second generation of CAR macrophages (CAR-Ms). We elucidated the role of the proprotein convertase furin in macrophages, demonstrating its overexpression in the presence of tumor cells. Importantly, furin inhibition maintains a proinflammatory macrophage phenotype, potentially redirecting them towards an antitumor state. Compared to furin-expressing counterparts, furin-inhibited CAR-Ms exhibited heightened antitumor phagocytic activity against breast cancer cells and ex vivo patient-derived tumoroids. Notably, they sustained a persistent proinflammatory profile, indicative of enhanced tumoricidal potential. Additionally, furin-inhibited CAR-Ms secreted factors that promote T-cell activation, offering a means to modulate the tumor microenvironment. In summary, our work highlights the translational potential of furin-inhibited CAR-Ms as a potent cellular therapy to mitigate macrophage exhaustion within the tumor environment. By capitalizing on macrophage-mediated antitumor responses, these findings pave the way for the development of second-generation CAR-M therapeutic strategies tailored for solid tumors.

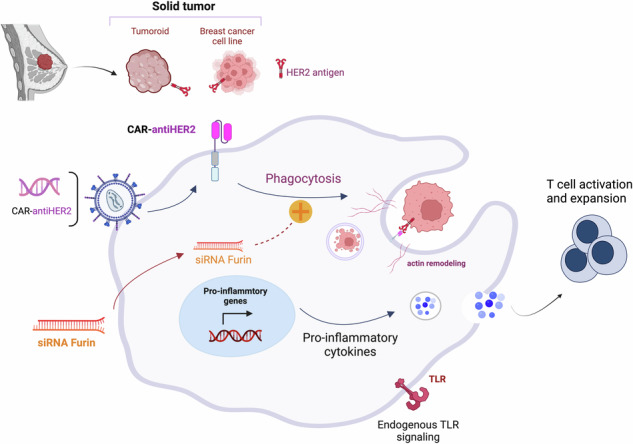

## Introduction

CAR cell therapy has revolutionized the treatment of hematological cancers [1]. However, this therapy remains ineffective in the context of solid tumors due to a number of physical and biological constraints. The main effector cells used in CAR therapies to date are T lymphocytes. In solid tumors, T lymphocytes have difficulty infiltrating dense and complex microenvironments and are sensitive to local immunosuppression, which reduces their antitumor activity. To overcome these obstacles, alternative immune effector cells that can be engineered to express a CAR receptor, such as CAR macrophages (CAR-Ms), are being investigated [2]. Macrophages are innate immune cells with phagocytic activity at the interface between innate and adaptive immunity. The advantage of utilizing macrophages over utilizing T cells is that they have a natural ability to infiltrate the tumor microenvironment (TME). In fact, macrophages can account for up to 50% of immune cells in the TME and are the most common type of immune cell in many cancers [3, 4]. To date, only a few clinical trials assessing the efficacy of CAR-Ms in the treatment of solid tumors are ongoing. The most advanced clinical trial consists of evaluating the antitumor activity of CAR-Ms targeting the HER2 antigen in solid tumors (NCT04660929) [5].

In very simple terms, macrophages are often defined according to their M1 (classical-activated macrophages) or M2 (alternative-activated macrophages) activation state. M1 macrophages exhibit antitumor activity through phagocytosis, the presentation of tumor antigens, and the release of soluble proinflammatory factors that stimulate the antitumor activity of other effector immune cells. Once in contact with the tumor, macrophages are rapidly converted to an M2 phenotype with pro-tumor activity. Finding a way to redirect the phenotype of macrophages toward an M1 phenotype is therefore crucial for maintaining their antitumor activity. This may also be a strategy to enhance the activity of CAR-Ms. In this regard, one study has shown that the Ad5f35 adenovirus, which mediates the transduction of primary macrophages, can also be involved in maintaining the M1 phenotype [6].

On the other hand, the involvement of proprotein convertases (PCs) in the regulation of the macrophage phenotype makes them good candidates for reprogramming for cancer therapy [7]. PCs are proteases involved in the processing of a variety of protein precursors, including proteases, cytokines, growth factors, and receptors. Nine PC genes are known: PC1/3, PC2, furin, PC4, PC5/6, PACE4, PC7, SKI-1/S1P and PCSK9 [8]. Furin and PC1/3 are the two PCs known to regulate the phenotype of immune cells. Furin is known to modulate the adaptive immune response of T cells; its inhibition leads to an imbalance in the Th1/Th2 polarization states [9, 10]. In addition, mice genetically engineered not to express furin in myeloid cells have elevated serum levels of proinflammatory cytokines due to an increase in the number of proinflammatory macrophages [11]. These furin-deficient macrophages overexpress many proinflammatory genes. We have further shown that PC1/3 also plays a role in regulating the macrophage phenotype [12–16]. PC1/3 affects the signaling pathways of several TLR receptors, and its inhibition leads to increased activation of these pathways, resulting in the activation of transcription factors that enable the expression of several proinflammatory cytokines [12, 13]. These studies therefore demonstrate the value of inhibiting these enzymes to reactivate macrophages, which could be used as part of a novel anticancer therapy. Several studies have demonstrated the potential of using an inhibitor of these enzymes as an immunotherapy [15, 17, 18]. The study by Tome et al. showed that the inhibitor reduced PD1 expression in T lymphocytes [17], while the studies by Rose et al. demonstrated the activation of tumor macrophages in a glioma model treated with a PC inhibitor [15, 18]. In addition, the FANG vaccine clinical trial, in which an autologous tumor-based product targeting furin by shRNAi DNA was found to be beneficial in patients with advanced cancer by boosting the antitumor immune response [19].

In this study, aiming to enhance the antitumor activity of CAR-Ms, we developed a combination therapy by employing CAR-Ms and PC inhibition to test whether PC inhibition could (1) maintain the proinflammatory M1 phenotype of macrophages in the TME and (2) enhance the phagocytic activity of CAR-Ms.

## Materials and Methods

### Data and resource availability

#### Material availability

The data generated in this study, including MS raw files, MaxQuant and DIA-NN files, and annotated MS/MS datasets, have been deposited with the ProteomeXchange Consortium via the PRIDE partner repository under accession number PXD058491.

### Experimental model and subject details

#### Tissue collection from human patients

The tumoroids were generated from human breast tumor tissue. All human breast tumor tissues were obtained from different patients who underwent surgery for early breast cancer. Fresh tumor tissue was obtained by a pathologist for tumoroid culture. The sample was anonymized before transfer to the laboratory. The study was approved by the local research committee of the Oscar Lambret Cancer Center and a French Ethical Committee (study IdRCB 2021-A00670-41). Written informed consent for the study was obtained from the patients before each procedure. All methods were performed in accordance with the relevant guidelines and regulations.

#### Purification of blood PBMCs

Peripheral blood mononuclear cells (PBMCs) were isolated by Ficoll density gradient centrifugation (Cytiva, France) from venous blood samples (EDTA tubes) obtained from healthy donors. Blood was obtained from the French Blood Establishment (EFS, Lille, France). All blood samples were processed within 24 hours of collection.

### Method details

#### Cell culture

The THP-1 and AU565 cell lines were cultured in RPMI medium supplemented with 10% FBS, 1% L-glutamine (2 mM), and 1% penicillin/streptomycin (100 units/ml). MDA-MB-231 cells were cultured in DMEM supplemented with 10% FBS, 1% L-glutamine (2 mM), and 1% penicillin/streptomycin (100 units/ml). All cell lines were cultured in a humidified atmosphere at 37 °C with 5% CO_2_.

### Isolation of human PBMCs and differentiation of macrophages

Human blood was collected from healthy adult donors by the Etablissement Français du Sang (EFS) and processed to isolate peripheral blood mononuclear cells (PBMCs) by Ficoll density gradient separation. Blood was diluted in 0.1% PBS-EDTA and gently layered on Ficoll (Sigma 1077, Ficoll-Paque PLUS, GE Healthcare density 1.077 g/ml). After centrifugation, the leukocyte ring containing monocytes was collected and washed in PBS-EDTA by centrifugation. The pellet was resuspended in red blood cell lysis buffer (eBioscience™ 10X RBC Lysis Buffer, Invitrogen) and incubated for 5 minutes at room temperature. After cell counting, the PBMCs were cultured in serum-free RPMI medium (RPMI 1640, 1% penicillin/streptomycin, and 2 mM L-glutamine) at 37 °C for 90 minutes. Nonadherent cells were removed, and adherent monocytes were cultured for seven days in RPMI 1640 medium supplemented with 10% fetal bovine serum, 1% penicillin/streptomycin, 2 mM L-glutamine, and 50 ng/mL M-CSF for differentiation into macrophages. For T-cell purification, PBMCs were labeled with microbeads using a pan T-cell isolation kit and separated on magnetic columns with an LS column separator, following the manufacturer’s recommendations (Miltenyi Biotec).

### Transfection of THP-1 cells and primary macrophages with siRNA

THP-1 cells were seeded at 500,000 cells per well in a 12-well plate and transfected with control siRNAs, furin siRNAs, or PC1/3 siRNAs at a final concentration of 50 nM. A Viromer® GREEN transfection kit (Lipocalyx) was used for the transfection reaction. Briefly, 0.5 µl of Viromer was mixed with 45 µl of Viromer buffer. This mixture was then added to 5 µl of siRNA previously diluted to 11 µM and incubated for 15 minutes at room temperature before being added to THP-1 cells. The THP-1 cells were then incubated with the transfection solution for 6 hours at 37 °C and differentiated into macrophages with 10 ng/ml phorbol 12-myristate 13-acetate overnight in a fresh medium (PMA Sigma Aldrich).

Primary macrophages were seeded at 600,000 cells per well in a 12-well plate and transfected with control siRNA or furin siRNA at a final concentration of 300 nM. An INTERFERin® transfection kit (Polyplus Transfection) was used for the transfection reaction. The siRNAs were diluted to a final concentration of 50 nM in Opti-MEM (Gibco, Life Technologies) and mixed with 12 µl of Interferin. This mixture was incubated for 10 minutes at room temperature and then added to the cells. The macrophages were incubated at 37°C for 24 hours. After 24 hours, the medium was changed to complete RPMI medium.

### Plasmid construction and virus

The CAR sequence was composed of a CD8a signaling sequence, the sequences of the variable parts of the light and heavy chains of trastuzumab to target the HER2 protein, a linker, a hinge sequence, a CD8a transmembrane domain and the intracytoplasmic region, corresponding to the ζ chain of the TCR/CD3 complex. This sequence of 6571 base pairs was inserted into a lentiviral vector or an adenoviral vector (pAd5/F35) under the control of the EF1a promoter. The coding sequence for the fluorescent protein EGFP was also present under the control of the CMV promoter. The control vector did not contain the CAR receptor sequence (mock). The vectors and the production of the viral particles (lentivirus and adenovirus) were performed by Vector Builder.

### Viral transductions

THP-1 cells were seeded in 24-well plates at 500000 cells per well. The cells were transduced using a lentiviral system at a multiplicity of infection (MOI) of 10 lentiviral particles per THP-1 cell in the presence of 8 μg/ml polybrene (Sigma Aldrich) in a complete medium. The plate was centrifuged at 1000 × *g* for 60 minutes at 32 °C to allow contact between the cells and the viral particles. Finally, the cells were pelleted by centrifugation, and the supernatant was replaced with a complete medium. THP-1 monocytes were sorted to obtain GFP+ cells that had integrated the vector. Cells were selected using a GFP fluorescence bandpass filter at 525/50 nm with a 488 nm laser on an SH800 cell sorter (Sony, Inc.). Monocytes were differentiated into macrophages in the presence of 100 ng/ml PMA in a complete medium. THP-1 cells were harvested 48 hours after differentiation and analyzed by flow cytometry and immunofluorescence to determine CAR expression.

Primary macrophages were transduced with an adenoviral system on day 5 after differentiation at an MOI of 200 based on the PFU titer. Differentiated macrophages were harvested on day 9 (96 hours post-transduction) and analyzed by flow cytometry and immunofluorescence to determine CAR expression.

### RT‒qPCR

Total RNA was extracted using TRIzol reagent (QIAGEN). A 1/5 volume of chloroform was then added, and the mixture was mixed. The mixture was then incubated for 15 minutes and centrifuged at 12,000 × *g* for 15 minutes at 4°C. The aqueous phase was transferred to a new tube, and an equal volume of isopropanol was added. The mixture was centrifuged at 12,000 × *g* for 10 min at 4°C. The supernatant was discarded, and the pellet was resuspended in 75% ethanol and centrifuged at 7500 × *g* for 5 min at 4°C. The supernatant was then thoroughly removed and discarded. The pellet was resuspended in 10 μl of nuclease-free water. Reverse transcription was further performed using Transcriptase Reverse SuperScript® III (Invitrogen, ThermoFisher Scientific) with 1 µg of total RNA as input. qRT‒PCR was performed using SYBR qPCR Mix (Applied Biosystems, ThermoFisher Scientific) according to the manufacturer’s protocol. A “negative RT” without the addition of reverse transcriptase was also included as a control to assess gDNA contamination. RT‒PCR was also performed using Go Taq polymerase according to the manufacturer’s protocol. The sequences of primers used are listed in Supplementary Fig. 1. The data are presented as the means ± standard errors of triplicate samples. Statistical significance between different siRNA conditions was calculated using ANOVA with multiple comparisons; ****P < 0.0001; ***P < 0.001; **P < 0.01; *P < 0.05; NS, not significant.

### Flow cytometry

The transduction efficiency of THP-1 cells and primary macrophages was determined by flow cytometry based on GFP expression. Mock, CAR-HER2 and untransduced (WT) cells were labeled with the Live Dead Near viability staining kit (633 nm, InvitrogenTM) and then washed and fixed with 4% paraformaldehyde (PFA). Transduction efficiency was determined as the percentage of GFP+ cells among live cells. CAR-HER2 expression in THP-1 cells and primary macrophages was tested using a two-step labeling protocol: binding of a biotinylated recombinant HER2 protein (ACROBiosystems) for 1 hour at 4 °C, followed by labeling with an anti-biotin PE antibody diluted 1:100 (Miltenyi Biotec) for 10 minutes at 4 °C. The cells were labeled with the viability marker Live Dead Near (633 nm, InvitrogenTM) for 20 minutes at 4 °C and then washed and fixed with 4% PFA. The number of macrophages expressing the CAR receptor was determined as the percentage of GFP + PE+ cells among live cells.

To study macrophage polarization, macrophages were inhibited with siRNAs (siFurin and siCTRL) as described above. After 48 hours of transfection, the macrophages were harvested and stained with fluorescein PC5-conjugated anti-human CD80 and BV786-conjugated anti-human CD86 (BD Biosciences).

To study lymphocyte activation, the cells were recovered after co-culture with tumoroids and macrophages. The cells were labeled with the following antibodies: Human anti-CD3 V450, Human anti-CD25 BV786, and Human anti-HLA-DR PE-Cy7 (BD Biosciences).

To determine HER2 expression in patient-derived tumoroids, they were dissociated with TrypLE for 15 minutes at 37 °C. The cells were washed and counted at a concentration of 500000 cells/condition. The cells were incubated with a PE-conjugated anti-human HER-2/neu antibody (BD Biosciences).

All the samples were analyzed using a CytoFLEX LX 2020 (Beckman Coulter). Isotype controls were included in all experiments. Flow cytometry data were processed using KALUZA software. The data are presented as the means ± standard errors of triplicate samples. Statistical significance between conditions was calculated using a t test; ****P < 0.0001; ***P < 0.001; **P < 0.01; *P < 0.05; NS, not significant.

### Immunofluorescence

100,000 mock or CAR-HER2 macrophages were seeded on glass coverslips pretreated with poly-D-lysine (Sigma Aldrich) in a 24-well plate. 1 µg of HER2-HisTag protein (200 µg/ml, CliniSciences) was added to the cells and incubated for 1 hour. The cells were then fixed with 4% PFA at 4 °C for 10 minutes. The slides were recovered and incubated overnight with a primary mouse anti-histidine antibody (1:100, R&D systems). The cells were then subsequently incubated with a secondary anti-mouse Alexa Fluor 647 antibody (1:200, ThermoFisher), followed by nuclear labeling with Hoechst (1/10,000). Slides were mounted on Faramount Dako mounting medium. Analysis was performed using a Zeiss LSM 510 confocal microscope coupled with a Zeiss Axiovert 200 M with a 63 × 1.4 numerical aperture oil immersion objective. ImageJ software was used for image processing.

### Migration and invasion assays

THP-1 cells were seeded at 240,000 cells/well in a 24-well plate. THP-1 cells were transfected with siRNAs (siFurin, siPC1/3, and siCTRL) as described previously. After 48 hours of transfection, the THP-1 cells were washed with DMEM. For the migration assay, 10,000 MDA-MB-231 cells were added to the transwell (8 µM), and for the invasion assay, the 8 µm transwell was coated with Matrigel before 200,000 MDA-MB-231 cells were added. The transwells were then placed in the THP-1 wells and incubated for 24 hours. T-cell chemotaxis was performed using a 48-well microchemotaxis Boyden chamber with 3-μm pore polycarbonate filters. The cells were incubated for 2 hours and 30 minutes with the secretome from the different co-culture conditions (Mock, CAR-HER2, CAR-HER2-siFurin). The inserts were collected, and the cells were fixed with 4% PFA, followed by nuclear labeling with Hoechst (1/10,000) for 20 minutes. The inserts were mounted on Faramount Dako mounting medium. Analysis was performed using a fluorescence microscope (Nikon). Five random fields per slide were photographed. ImageJ software was used for image processing. The data are presented as the means ± standard errors of triplicate samples. Statistical significance between different siRNA conditions was calculated using ANOVA with multiple comparisons; ****P < 0.0001; ***P < 0.001; **P < 0.01; *P < 0.05; NS, not significant.

### MTS cell proliferation assay

T cells were isolated as previously described, and 100,000 T cells were seeded into a 96-well plate with the secretome from the various co-culture conditions (Mock, CAR-HER2, CAR-HER2-siFurin). The MTS assay was conducted according to the manufacturer’s instructions (Promega).

### Bead-based phagocytosis assay

A total of 100000 mock or CAR-HER2 macrophages were seeded on glass coverslips pretreated with poly-D-lysine (Sigma Aldrich) in a 24-well plate. Furin and PC1/3 inhibition was performed as described above. Monocytes were differentiated into macrophages with 100 ng/ml PMA overnight.

Streptavidin-coated polystyrene microparticles (5.0–5.9 μm diameter, Spherotech) were sterilized in 70% isopropanol for 20 min. The beads were then incubated with the fluorescent dye pHrodo SE (Invitrogen) diluted in sodium bicarbonate buffer (0.1 M, pH 8.5) at a concentration of 10 μmol/l for 30 min in the dark. After washing, half of the beads were incubated with biotinylated HER2 protein (ACROBiosystems) at a concentration of 2.5 μg per mg of beads in phosphate buffer (0.1 M, pH 6.5) for 1 h. The other half was used as a blank bead control (not bound to the biotinylated HER2 protein). After washing, the beads were placed in the presence of mock or CAR macrophages at a ratio of 10 beads per cell for 4 h. The cells were then fixed with 4% PFA at 4 °C for 10 min, followed by nuclear labeling with Hoechst (1/10,000). Slides were mounted on Faramount Dako mounting medium. Analysis was performed using a Zeiss LSM 510 confocal microscope coupled to a Zeiss Axiovert 200 M with a 63 × 1.4 numerical aperture oil immersion objective. Five random fields per slide were photographed. ImageJ software was used for image processing. Phagocytosis efficiency was determined by the proportion of cells phagocytosing at least one bead out of the total number of macrophages. The data are presented as the means ± standard errors of triplicate samples. Statistical significance between different conditions was calculated using ANOVA with multiple comparisons; ****P < 0.0001; ***P < 0.001; **P < 0.01; *P < 0.05; NS, not significant.

### Flow cytometry-based phagocytosis assay

THP-1 mock or THP-1 CAR-HER2 cells were cocultured with AU565 (HER2+) or MDA-MB-231 (HER2-) tumor target cells at a ratio of 1/1, 1/5 or 1/10 (target/effector) for 6, 24 or 48 h at 37 °C. Cancer cells were prelabeled with Cytotell blue (AAT Bioquest) at a 1:500 dilution for 20 min at 37 °C prior to coculture.

Similarly, mock or CAR-HER2 human monocyte-derived macrophages (96 h post-transduction) were cocultured with AU565 cells (HER2+) or MDA-MB-231 (HER2-) tumor target cells at a 3:1 (effector/target) ratio for 6 or 24 hours at 37 °C. CAR-HER2 cells were transfected with a control siRNA or a furin siRNA (as previously described). Tumor cells were prelabeled with Cytotell blue (AAT Bioquest) as described above. After coculture, the cells were trypsinized and analyzed by FACS using a CytoFLEX LX 2020 (Beckman Coulter). The percentage of BFP + GFP+ events was reported as the percentage of phagocytosis. The data are presented as the means ± standard errors of triplicate samples. Statistical significance between Mock, CAR-HER2-siCtrl and CAR-HER2-siFurin was calculated using ANOVA; ****P < 0.0001; ***P < 0.001; **P < 0.01; *P < 0.05; NS, not significant.

### Microscopy-based phagocytosis assay

A total of 200000 human monocyte-derived macrophages were seeded on 14 mm coverslips. Cells were transduced with either CAR-HER2 or empty vector (mock). At 96 hours post-transduction, CAR-HER2 macrophages were transfected with siRNA control or siRNA furin. After 48 hours of transfection, Mock, CAR-HER2-siCtrl, or CAR-HER2-siFurin cells were cocultured with AU565 or SKBR3 tumor target cells at a ratio of 3:1 (effector/target) for 6 hours at 37 °C. The cancer cells were pre-labeled with CytotellBlue (1/500). The coverslips were collected, and the cells were fixed with 4% PFA for 10 minutes at 4 °C, followed by nuclear labeling with SYTOX^TM^ Deep Red Nuclei Acid Stain (1/2000). Slides were mounted with Faramount Dako mounting medium. Analysis was performed using a Zeiss LSM 510 confocal microscope coupled to a Zeiss Axiovert 200 M with a 63 × 1.4 numerical aperture oil immersion objective. Five random fields per slide were photographed. ImageJ software was used for image processing.

### Cytokine array

A Human Cytokine Antibody Array (ab133997, Abcam) was used to quantify 42 cytokines in a culture medium according to the manufacturer’s instructions. The medium was obtained from primary macrophages that were mock, CAR-HER2, or CAR-HER2-transfected with siFurin.

After the array membranes were incubated in 1× blocking buffer for 30 min at room temperature, a sample of each culture medium was applied to the membranes and incubated overnight at 4 °C on a rocking platform shaker. After four washes in Wash Buffer I and three washes in Wash Buffer II, the membranes were incubated overnight at 4 °C with biotin-conjugated anti-cytokines and then with HRP-conjugated streptavidin. The washed arrays were then treated with chemiluminescence detection reagents, and images were captured using an iBright CL750 Imaging System (Invitrogen). The levels of each cytokine were quantified using Fiji software. The data are presented as the means ± standard errors of triplicate samples. Statistical significance between Mock, CAR-HER2-siCtrl and CAR-HER2-siFurin was calculated using ANOVA with multiple comparisons; ****P < 0.0001; ***P < 0.001; **P < 0.01; *P < 0.05; NS, not significant.

### CAR-M cell sorting by FACS after coculture

After 24 hours of coculture of primary CAR-HER2 or mock macrophages with AU565 tumor cells at a ratio of 3:1, the cells were trypsinized and sorted. Primary CAR-HER2 macrophages were selected using a GFP fluorescence bandpass filter at 525/50 nm with a 488 nm laser on an SH800 cell sorter (Sony, Inc.).

### Protein extraction and Western Blot analysis

200,000 primary macrophages were seeded in 6-well plates and transfected with either siRNA control or siRNA Furin, as previously described. The cells were collected, washed once with ice-cold PBS, and lysed with RIPA buffer for total protein extraction, which was quantified using the Bradford method. Total cell extracts (20 μg) were analyzed by Western blotting. Proteins were separated by SDS-PAGE and transferred onto a nitrocellulose membrane. Membranes were blocked for 1 hour at room temperature in TBS-Tween (0.1%) supplemented with 5% milk and incubated overnight at 4 °C with rabbit anti-phospho-IκBα, rabbit anti-IκBα, rabbit anti-phospho-STAT3, and rabbit-anti-STAT3 (1:1000, from Cell Signaling Technology, Leiden, The Netherlands). Horseradish peroxidase-coupled goat anti-rabbit was used at 1:20,000. Proteins were visualized with the enhanced chemiluminescence kit (West Dura, Pierce) according to the manufacturer’s instructions. Images were captured using the iBright CL750 Imaging System (Invitrogen).

### Proteomic sample preparation

THP-1 cells were transfected with furin siRNA, PC1/3 siRNA, or the siRNA control (as described previously). After 48 hours of transfection, proteins were extracted with RIPA buffer and quantified by the Bradford method. Thirty micrograms of protein cell extracts were reduced with an equivalent volume of reducing buffer (dithiothreitol-DTT 0.1 M) for 40 minutes at 56 °C and loaded into Amicon ultracentrifugal filters (Millipore). The samples were then subjected to the filter-aided sample preparation (FASP) protocol, which included denaturation in urea buffer (8 M Urea, 0.1 M Tris-HCl, pH 8) and alkylation with 55 mM iodoacetamide for 20 minutes in the dark. The proteins were then digested overnight at 37 °C with 40 µg/ml trypsin (Promega). Digestion was stopped with 0.5% TFA. The samples were desalted using a Millipore C18 ZipTip or an Evotip Pure (Evosep, Denmark) and eluted with 20 µl of elution solution (80% ACN/20% 0.1% TFA). The solution was then dried with a SpeedVac. Dried samples were solubilized in a resuspension solution (2% ACN/80% formic acid 0.1%) prior to LC-MS/MS analysis. Similarly, after cell sorting, proteins were extracted and digested as described above.

### LC-MS/MS analysis

The samples were separated either by on-line reversed-phase chromatography using a Thermo Scientific Easy-nLC 1000 system equipped with a trap column (75 µm ID × 2 cm, Thermo Scientific) and a C18 packed-tip column (75 µm ID × 50 cm, Thermo Scientific). Peptides were separated using increasing amounts of acetonitrile (5–35% over 100 min) at a flow rate of 300 nl/min. The LC eluent was electrosprayed directly from the analytical column, and a voltage of 2.4 kV was applied via the liquid junction of the nanospray source. The chromatography system was coupled to a Thermo Scientific Q Exactive mass spectrometer, which was programmed to acquire data in a data-dependent acquisition mode (top 10). Full scan MS analysis was performed over a m/z range of 300 to 1600, a resolution of 70,000 FWHM, an AGC of 3e6 ions, and a maximum injection time of 120 ms. For MS/MS analysis, the m/z mass range was set between 200 and 2000, with an AGC of 5e4 ions, a maximum injection time of 60 ms, and a resolution of 17,500 FWHM. The higher energy collision dissociation (HCD) was set to 30%. Precursor ions with charge states greater than +1 and less than +8 were selected for fragmentation, with a dynamic exclusion time of 25 seconds.

For the lymphocytes, the peptides were loaded and analyzed by Evosep One LC system coupled to a timsTOF HT (Bruker Daltonics, Germany) mass spectrometer. The Evosep One system operated with the 60 Samples Per Day (60 SPD) method using the C18 performance column (EV1109; 8 cm × 150 μm, 1.5 μm) maintained at 40 °C. The analytical column was connected using a fused silica ID emitter (10 μm ID; Bruker Daltonics) within a Captive spray source (Bruker). Samples were acquired in dia-PASEF mode, with spectra acquired within an m/z range of 100–1700 and an Ion Mobility range from 1/K_0_ = 1.51 V cm^−2^ to 1/K_0_ = 0.6 V cm−2.

### Data analysis

Macrophage proteins were identified using MaxQuant software [20] version 1.6.10.43 by comparing all MS/MS data with the proteome database of the complete reviewed proteome of *Homo sapiens* (UniProt, release July 2018; 8054 entries). Lys-C trypsin specificity was used for the digestion mode with two missed cleavages. Cysteine carbamidomethylation was set as a fixed modification. N-terminal acetylation and methionine oxidation were chosen as variable modifications. For the MS spectra, an initial mass tolerance of 6 ppm was selected, and the MS/MS tolerance was set to 20 ppm for the HCD data. For identification, the false discovery rate (FDR) was set at 0.01 for peptide spectral matches (PSMs) and at the protein level. Relative, label-free quantification of proteins was performed using the MaxLFQ algorithm integrated into MaxQuant with the default parameters [21]. Analysis of the identified proteins was performed using Perseus software (http://www.perseus-framew ork.org/) (version 1.6.10.43) [22]. The file containing the identification information was used with hits to the reverse database, and proteins identified with modified peptides and potential contaminants were removed. The LFQ intensity was then logarithmized (log2[x]). Categorical annotation of the rows was used to define different groups (CAR-HER2 and CAR-HER2-siFurin). Two-sample *t*-test was performed using Student’s *t*-test with a *p* value of 0.05, with grouping maintained during randomization. The results were normalized to the z scores and are presented as hierarchical clustering. Functional annotation and characterization of the identified proteins were performed using STRING (version 10.5, http://string-db.org) and Funrich (version 3.1.3, http://www.funrich.org/).

The proteomics data analysis for lymphocytes analysis was conducted using DIA-NN software (version 1.8.1). A search against the human UniProt-reviewed Homo sapiens database (downloaded in May 2023, 20,422 entries) was performed using library-free workflow. A maximum of 2 trypsin missed cleavages were allowed and the maximum variable modification was set to 3. Carbamidomethylation (Cys) was set as the fixed modification, whereas methionine oxidation was set as the variable modification. The peptide length range was set to 7–30 amino acids, precursor charge range 2–4, precursor m/z range 300–1300, and fragment ion m/z range 100–1700. False discovery rates (FDRs) at the protein and peptide level were set to 1%. Match between runs was allowed and “robust LC (high accuracy)” as the quantification strategy was enabled. Analysis of the identified proteins was performed using Perseus (version 1.6.10.43) software. Statistical tests were performed using ANOVA with a false discovery rate (FDR) of 1%. Visual representation of significant protein variations was obtained using hierarchical clustering analysis in the form of a heatmap.

### Subnetwork enrichment pathway analysis

Elsevier’s Pathway Studio (version 11.0//Elsevier) was used to map all relationships between the differentially expressed proteins across all conditions based on the Ariadne ResNet. For proteins identified in the shotgun analysis, the subnetwork enrichment analysis (SNEA) algorithm was used to detect the significantly altered biological pathways in which the identified proteins were involved. This algorithm uses Fisher’s statistical test to detect any nonrandom associations between two categorical variables organized by a specific relationship. This algorithm also starts by creating a central “seed” from all the relevant identities in the database and establishes links to associated entities based on their relationship to the seed. SNEA compares the subnetwork distribution to the background distribution using a one-sided Mann–Whitney U test and calculates a *p*-value, which represents the statistical significance between different distributions. In all analyses that we performed, the GenBank ID was used to create experimental groups based on the different conditions used for analysis. Pathway networks were constructed based on biological processes and molecular functions for each individual protein, together with their associated targets.

### Primary breast cancer tumoroid culture and coculture with macrophages

The tumor tissue biopsy sample was minced and placed in digestion medium in a reduced volume of 2 mL to avoid loss of material. The digestion medium consisted of Hank’s balanced salt solution (HBSS, Gibco) with antibiotics and antifungals (1x penicillin/streptomycin, 1x amphoteromicin) containing 1 mg/mL collagenase type IV (Sigma) and 5 U/mL hyaluronidase (Sigma). The tumor tissue was digested at 37°C for 2 hours and mixed every 15 minutes to facilitate digestion. After digestion, 6 mL of HBSS with antibiotics was added, and the cell suspension was filtered through a 100 μm filter (Dutcher) to retain residual tissue pieces. The suspension was centrifuged at 300 × *g* for 5 minutes. If a red pellet was visible, the erythrocytes were lysed in 1 mL of red blood cell lysis buffer (RBC, Invitrogen) for 5 minutes at room temperature. The suspension was then mixed with 6 mL of HBSS supplemented with antibiotics and centrifuged at 300 × *g* for 5 minutes. The cell pellet was resuspended in a reduced growth factor-soluble basement membrane matrix for organoid culture (Matrigel®, Corning) and plated dropwise into 24-well plates. The Matrigel was allowed to solidify in the incubator for 30 minutes, after which 500 μL of complete culture medium was added. The complete tumoroid culture medium consisted of Advanced DMEM (Gibco) supplemented with 1X GlutaMAX, 10 mM HEPES, 1X penicillin/streptomycin, 1X amphoteromicin, 50 μg/mL Primocin, 1X B27 supplement, 5 mM nicotinamide, 1.25 mM N-acetylcysteine, 250 ng/mL R-spondin 1, 5 nM heregulinβ-1, 100 ng/mL noggin, 20 ng/mL FGF-10, 5 ng/mL FGF-7, 5 ng/mL EGF, 500 nM A83-01, 500 nM SB202190 and 5 μM Y-27632. Semi-liquid coculture was performed in non-adherent PrimeSurface plates (MS-9024OZ) in which mock or CAR macrophages (siControl or siFurin) were mixed with tumoroids (HER2+ or HER2-) in a complete tumoroid culture medium supplemented with 2% Matrigel at a 3:1 (effector/target) ratio for 48 hours at 37 °C. When T cells were added to the co-culture, they were introduced at a ratio 1:1 (lymphocyte:macrophage). The co-culture model was maintained for 24, 48, and 72 hours. At each time point, cells were collected for analysis by flow cytometry and proteomic analysis.

### Microscopy of tumoroids

After 48 hours of coculture, the tumoroids were collected and placed in a Labteck plate (Dutscher) previously coated with poly-D-lysine for 24 hours at 37 °C to allow adhesion. The mixed tumoroids were then fixed with 4% PFA for 24 hours at 4 °C, followed by permeabilization (with PBS, 1% Triton X-100, and 0.1% Tween+0.1 M glycine) for 24 hours at 4 °C. The nuclei were then labeled with Hoechst (1/2000) for 24 hours at 4 °C. Finally, a series of clearing steps were performed using a buffer consisting of 25% formamide and 10% polyethylene glycol for 1 hour, followed by 50% formamide and 20% polyethylene glycol for 6 hours. The clearing steps were performed at room temperature. Tumoroids were stored in a clearing buffer until analysis. Analysis was performed on a CSU-W1 spinning disc (Gataca Systems) with a Live-SR module controlled by Metamorph software. An initial analysis was performed using a 40x/1.3 numerical aperture oil immersion objective, followed by an analysis using a 10x/0.3 numerical aperture oil immersion objective. Image stacks were acquired with a delta z of 0.3 µm. Five random fields per condition were photographed. ImageJ software was used for image processing. Statistical significance between mock, CAR-HER2-siCtrl and CAR-HER2-siFurin was calculated using a t test; ****P < 0.0001; ***P < 0.001; **P < 0.01; *P < 0.05; NS, not significant.

### Flow cytometry-based tumoroid phagocytosis assay

Similarly, mock or CAR macrophages (siControl or siFurin) were cocultured with tumoroids (HER2+ or HER2-) in a complete tumoroid culture medium supplemented with 2% Matrigel at a 3:1 (effector/target) ratio for 48 hours at 37 °C. Tumoroids were prelabeled with Cytotell blue (AAT Bioquest) as described above. After coculture, the cells were harvested and analyzed by flow cytometry using a CytoFLEX LX 2020 (Beckman Coulter). The percentage of CytotellBlue+ events was reported as the percentage of phagocytosis. The percentage cytotoxicity was calculated as follows: % cytotoxicity = [(1 − (the remaining number of target cells from treated groups/the number of target cells alone)] × 100. The data are presented as the means ± standard errors of triplicate samples. Statistical significance between Mock, CAR-HER2-siCtrl and CAR-HER2-siFurin was calculated using ANOVA with multiple comparisons; ****P < 0.0001; ***P < 0.001; **P < 0.01; *P < 0.05; NS, not significant.

## Results

### Breast cancer influences the expression of proprotein convertase enzymes in macrophages, and inhibiting these enzymes promotes a proinflammatory profile

We investigated whether cancer cells could influence the expression of two PC enzymes, Furin and PC1/3, previously known to play a role in the phenotypic control of mouse and rat macrophages [11, 12, 23], in human THP-1 macrophages and primary blood monocyte-derived macrophages. First, basal expression of Furin and PC1/3 has previously been demonstrated in THP-1 macrophages [24, 25], and we validated the expression of Furin in primary macrophages by PCR (Supplementary Fig. 2). However, PC1/3 expression was not detected in the primary macrophages (Supplementary Fig. 2).

**Fig. 1:**
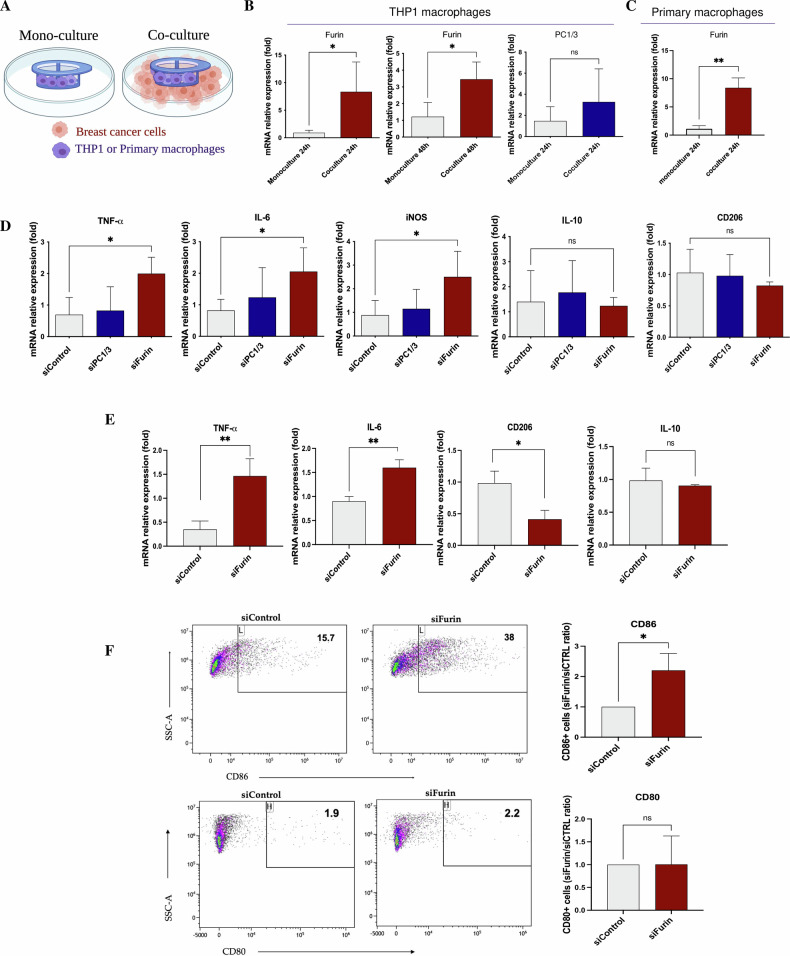
Inhibition of furin proprotein convertase induces the activation of THP-1 cells and primary human macrophages. **A** Illustration of coculture between THP-1 macrophages/primary macrophages and a breast cancer cell line. **B** Expression of PC1/3 and furin in THP-1 macrophages after 24 h and 48 h of coculture with cancer cells. The fold change is expressed relative to the mRNA levels obtained from cells grown in monoculture (*n* = 3). **C** Furin expression in primary macrophages after 24 h and 48 h of coculture with cancer cells. The fold change is expressed relative to the mRNA levels obtained from cells grown in monoculture (*n* = 3). **D** Expression of pro- and anti-inflammatory markers by THP-1 macrophages transfected with furin, PC1/3 or control siRNAs. TNF-α, IL-6, iNOS, IL-10 and CD206 mRNA levels were assessed by qPCR. The fold change is expressed relative to the mRNA levels obtained from cells treated with the siRNA control (*n* = 3). **E** Expression of pro- and anti-inflammatory markers in primary macrophages transfected with furin or control siRNAs. TNF-α, IL-6, IL-10 and CD206 mRNA levels were assessed by qPCR. The fold change is expressed relative to the mRNA levels obtained from cells treated with the siRNA control (*n* = 3). **F** Expression of the costimulatory molecules CD80 and CD86 was assessed by flow cytometry in primary macrophages transfected with furin and control siRNAs. Scatter plots showing the frequency of CD86+ and CD80+ macrophages. The graphs show the ratio of CD80+ and CD86+ siFurin macrophages relative to siControl macrophages (*n* = 3).

Using a transwell coculture system (Fig. 1A), we showed that furin expression increased in THP-1 cells after coculture with human breast cancer cells (Fig. 1B). Seven-fold and 3-fold increases were observed after 24 and 48 hours of coculture, respectively. This effect was also observed in primary macrophages (Fig. 1C), with a 7-fold increase after 24 hours of coculture. A slight increase in PC1/3 expression was observed in THP-1 cells after 24 hours of coculture, although the difference was not significant (Fig. 1B). These results suggest that to elicit immune suppression, cancer cells induce an increase in the expression of PC enzymes in macrophages. Indeed, the downregulation of Furin and PC1/3 has been reported to induce the proinflammatory activation of mouse and rat macrophages [11, 12, 23, 26]. To confirm that Furin and PC1/3 downregulation have the same effect on human macrophages, we knocked down Furin or PC1/3 by using a pool of small interfering RNAs (siRNAs) in THP-1 cells (Supplementary Fig. 3) and in primary macrophages (Supplementary Fig. 4). Macrophage activation status was assessed by measuring the mRNA expression of several pro- and anti-inflammatory markers by qPCR. An increase in the expression of proinflammatory markers was detected in THP-1 cells following Furin knockdown (Fig. 1D). A 2-fold increase in TNF-α, IL-6 and iNOS expression was observed. No difference in the expression of IL-10 or CD206, two anti-inflammatory markers, was detected. Slight but insignificant changes in the expression of these markers were induced by PC1/3 knockdown in THP-1 macrophages (Fig. 1D). In primary macrophages, a 1.5-fold increase in the expression of TNF-α and IL-6 was observed, while CD206 expression was reduced following furin knockdown (Fig. 1E). These results show that silencing furin in human macrophages can induce a proinflammatory phenotype, which could be a strategy to maintain the antitumor activities of macrophages.

Macrophage polarization was also assessed by flow cytometry by measuring the number of CD86+ and CD80+ cells, two proinflammatory markers. While no difference was observed in the number of CD80+ cells, a 2-fold increase in the number of CD86+ cells was observed after furin knockdown compared to that in control primary macrophages (Fig. 1F). This proinflammatory state is accompanied by antitumor effects, as inhibition of PC1/3 and Furin in macrophages prevents the migration and invasion of breast cancer cells (Supplementary Fig. 5).

**Fig. 2:**
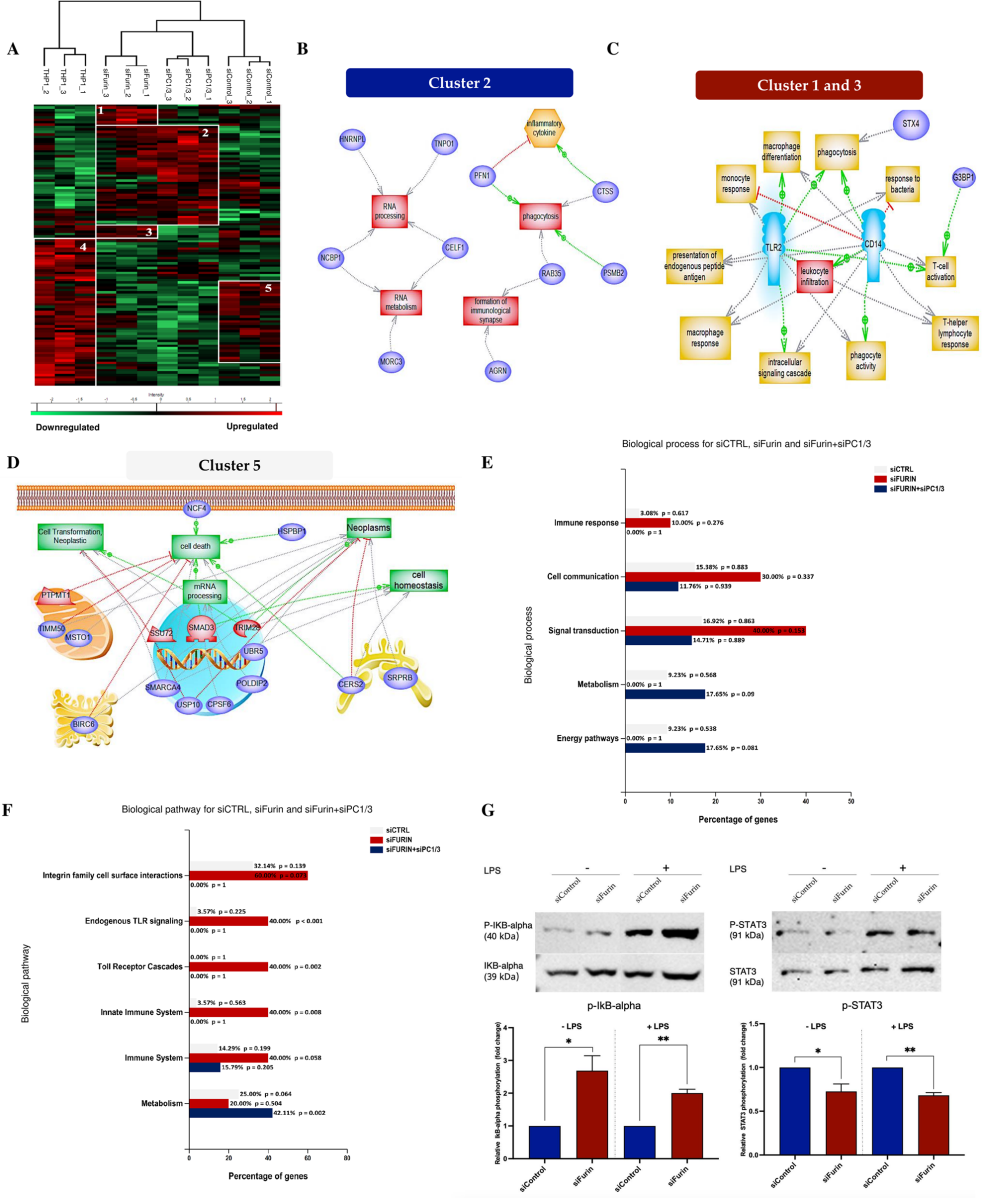
Furin- and PC1/3-inhibited THP-1 macrophages express intracellular proteins associated with the proinflammatory functions of macrophages. **A** THP-1 macrophages were transfected with furin, PC1/3, and control siRNAs (siCtrl). Cells were lysed prior to FASP and LC-MS/MS analysis. MaxQuant and Perseus software were used for the statistical analysis, and a heatmap was generated to show proteins that were significantly different between siCtrl, siFurin, siPC1/3, and THP-1 WT macrophages. Five clusters are highlighted (*n* = 3). **B**–**D** Global analysis of protein pathways expressed in THP-1 WT and THP-1 cells transfected with different siRNAs (siFurin, siPC1/3 or siCtrl). **E**, **F** Biological processes associated with the siControl, siFurin, and siFurin+siPC1/3 clusters. The analysis was performed using FunRich software. **G** Western blot analysis of phospho-IκB-α, total IκB-α, phospho-STAT3, and STAT3 in primary macrophages transfected with furin or control siRNAs, with or without LPS stimulation for 3 hours (200 ng/mL). Quantification of phospho-IκB-α and phospho-STAT3 levels is presented as the fold increase relative to control macrophages, normalized to total IκB-α and STAT3, respectively (*n* = 2).

We then investigated the effect of PC1/3 and furin inhibition on the macrophage proteome. After trypsin digestion, the proteins were analyzed by liquid chromatography coupled with mass spectrometry (LC‒MS). A total of 2393 proteins were identified (Supplementary Table 1). To identify proteins with the most prominent differences in expression profiles between transfected macrophages transfected with the siRNAs PC1/3 and furin compared to those transfected with the siRNA control (siCtrl) or untransfected macrophages, we used ANOVA with a false discovery rate (FDR) of 5%. A total of 123 differentially expressed proteins were identified (Supplementary Table 2). The significantly up- and downregulated proteins between conditions are shown on a heatmap (Fig. 2A). An enrichment analysis of the differentially expressed proteins was performed for each cluster indicated in the heatmap. The cluster of proteins upregulated in macrophages transfected with furin siRNA and PC1/3 siRNA (siFurin and siPC1/3, respectively, corresponding to cluster 2) was associated with RNA metabolism and the immune response (e.g., phagocytosis and the formation of immunological synapses; Fig. 2B, E and F). The cluster of proteins upregulated only in macrophages transfected with siRNA furin (corresponding to clusters 1 and 3) was associated with cell communication related to strong activation of the immune response (e.g., phagocytosis, macrophage response, T-cell activation, antigen presentation and Toll-like receptor signaling; Fig. 2C, E and F). Interestingly, the expression of TLR2 and CD14, which are coreceptors of several TLR receptors, increased after furin inhibition (Supplementary Table 2). Finally, the cluster of proteins upregulated only in macrophages transfected with the siRNA control (siControl, corresponding to cluster 5) was associated with signal transduction and metabolism (e.g., cell transformation, mRNA processing and neoplasia; Fig. 2D, E and F). Cluster 4 contained proteins overexpressed in nontransfected THP-1 macrophages. Most of the proteins were also expressed in the siRNA control-transfected macrophages (Fig. 2A), and the same biological pathways were involved (Supplementary Fig. 6).

We also analyzed the proteins that were only present in one condition. A total of 31, 20, and 41 proteins were specific for macrophages transfected with the siRNA control, siRNA furin, and siRNA PC1/3, respectively (Supplementary Fig. 7A and Supplementary Table 3). Enrichment analysis revealed an abundance of proteins related to cell communication and cell proliferation in macrophages transfected with the siRNA control (Supplementary Fig. 7A–C). Enrichment analysis of the proteins exclusive to siRNA furin-transfected macrophages confirmed the immune activation status of these cells, as reflected by their association with several processes involved in macrophage infiltration, monocyte activation, and interleukin signaling, with IL1B, for example, being found in this group of proteins (Supplementary Fig. 7A–C). Enrichment analysis of proteins exclusive to the siRNA PC1/3-transfected macrophages also revealed a stronger immune response (Supplementary Fig. 7A–C). Notably, processes such as cell communication and signal transduction exhibited significant enrichment under these conditions.

Western blot analysis was used to assess the impact of furin inhibition on macrophage activation, specifically by examining the phosphorylation of IκB-α as an indicator of NFκB transcription factor activity. Our results demonstrated that phosphorylation of IκB-α was elevated in furin-deficient macrophages, both in the presence and absence of LPS stimulation. Conversely, activation of the anti-inflammatory STAT3 pathway was reduced in furin-inhibited macrophages (Fig. 2G, Supplementary Fig. 8).

In conclusion, the proteome of macrophages was profoundly altered after PC1/3 or furin inhibition, with the most pronounced effect on the pro-inflammatory immune activation of these cells, as indicated by activation of the NFκB pathway. Since no PC1/3 expression was detected at the basal state in primary macrophages, and considering the effect of furin appears to play a more significant role in the phenotypic control of macrophages, even in THP-1 macrophages, we decided to focus on the role of furin in subsequent experiments. Moreover, since furin inhibition is associated with the enrichment of the phagocytosis pathway, it could enhance the activity of CAR-Ms.

### Furin silencing in CAR-Ms enhances their phagocytic activity

**Fig. 3:**
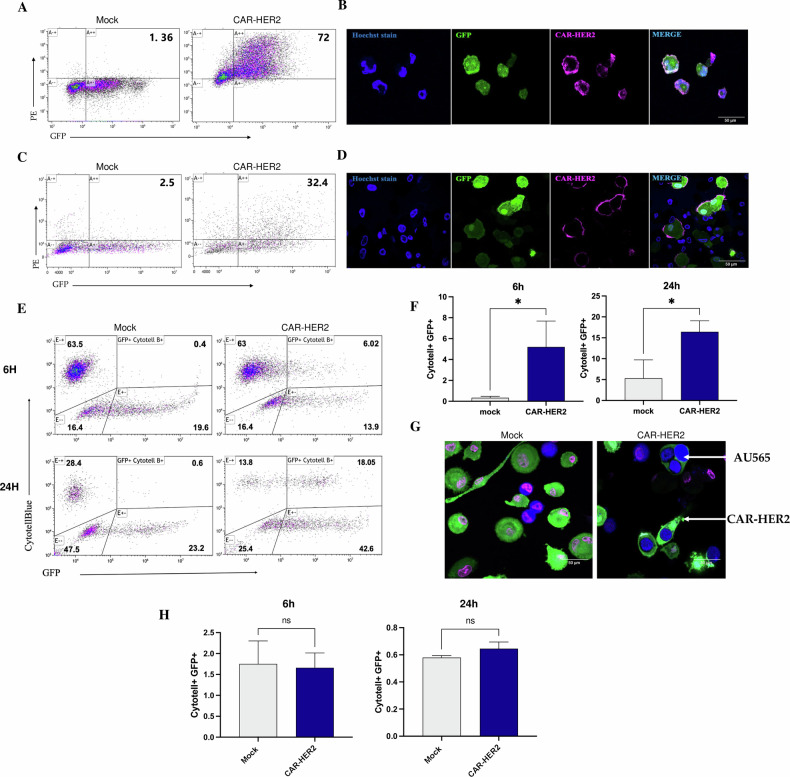
CAR macrophages show targeted antitumor activity. **A**–**C** Validation of CAR expression in THP-1 cells and primary macrophages by flow cytometry. Scatter plots showing the frequency of PE + GFP+ macrophages, which are macrophages expressing the CAR receptor. CAR receptor-expressing cells were revealed by incubation with biotinylated HER2 protein and an anti-biotin PE antibody. **B**–**D** Validation of CAR expression in THP-1 cells and primary macrophages by microscopy. Cells expressing the CAR receptor were detected by incubation with HER2-HisTag protein, and the CAR was detected by incubation with a primary anti-histidine antibody recognized by a secondary antibody Alexa Fluor 647. **E** Representative FACS plots of phagocytosis after 6 and 24 hours of coculture at a 3:1 (macrophage/cancer cell) ratio. The rate of phagocytosis was determined as the percentage of CytotellBlue+ and GFP+ cells among all cells. **F** Quantification of the phagocytosis of HER2+ cancer cells by mock and CAR-HER2 cells after 6 and 24 hours of coculture (*n* = 3). **G** Immunofluorescence analysis of the phagocytosis of AU565 (HER2+) cells by mock and CAR-HER2 macrophages. The macrophages were GFP+, the cancer cells were CytotellBlue+, and the nuclei were stained with SYTOX^TM^ Deep Red Nuclei Acid Stain. **H** Quantification of the phagocytosis of HER2- cancer cells by mock and CAR-HER2 cells after 6 and 24 hours of coculture (*n* = 3).

The application of CAR therapies to solid tumors remains challenging. Macrophages may be inhibited in an immunosuppressive tumor environment, and CAR-Ms may lose their antitumor activity. We developed CAR-Ms targeting the HER2 antigen, which is overexpressed in a subtype of breast cancer. Both THP-1 cells and human blood monocyte-derived macrophages were transduced with a first-generation anti-HER2 CAR construct with CD3ζ as the intracellular domain (Supplementary Fig. 9A). The construct also contained a GFP sequence. The construct was transduced into THP-1 macrophages using a lentiviral vector, resulting in more than 70% of the macrophages expressing the CAR receptor and GFP (Fig. 3A). Control (mock) macrophages were transduced with a construct containing only the GFP sequence (Fig. 3A). We also confirmed the expression of the CAR receptor on the surface of THP-1 macrophages by immunofluorescence (Fig. 3B). We showed that GFP expression was stable for at least 4 weeks (Supplementary Fig. 9B). In primary macrophages, the construct was transduced with an adenoviral vector, resulting in 30% of the macrophages expressing the CAR receptor and GFP (Fig. 3C). Mock cells were similarly transduced with an adenoviral vector lacking the CAR sequence. CAR receptor expression on the surface of primary macrophages was also confirmed by immunofluorescence (Fig. 3D). To investigate the functions of these anti-HER2 CAR-Ms, we cocultured the cells with either HER2+ or HER2- breast cancer cells to measure their phagocytic activity. CAR-THP-1 cells specifically phagocytized HER2+ cancer cells at every ratio tested after 24 h of coculture (as assessed by the number of cancer cells remaining after coculture, Supplementary Fig. 9C, D). No phagocytic activity was observed when CAR-THP-1 cells were cocultured with HER2- cancer cells (Supplementary Fig. 9C, D). Primary CAR-Ms or mock-generated macrophages were cocultured with HER2+ breast cancer cells (AU565) at a ratio of 3 effector cells to 1 target cell for 6 or 24 hours. Phagocytic activity was assessed by flow cytometry as the percentage of double-positive CAR-M GFP+ and AU565 CytotellBlue+ cells. After 6 hours of coculture with mock macrophages, the percentage of double-positive cells was approximately 0.5%, whereas more than 5% of CAR-Ms were GFP+ CytotellBlue+ (Fig. 3E). The difference was statistically significant (Fig. 3F). After 24 hours of coculture, the percentage of phagocytosis increased significantly between mock-treated macrophages and CAR-Ms (Fig. 3E, F). We also confirmed by immunofluorescence that the cancer cells were internalized by the CAR-Ms (Fig. 3G). No phagocytic activity was observed when CAR-M were co-cultured with HER2-negative cancer cells (Fig. 3H). Additionally, the phagocytic activity of CAR-M was verified in a separate HER2-positive breast cancer cell line (Supplementary Fig. 10A, B).

**Fig. 4:**
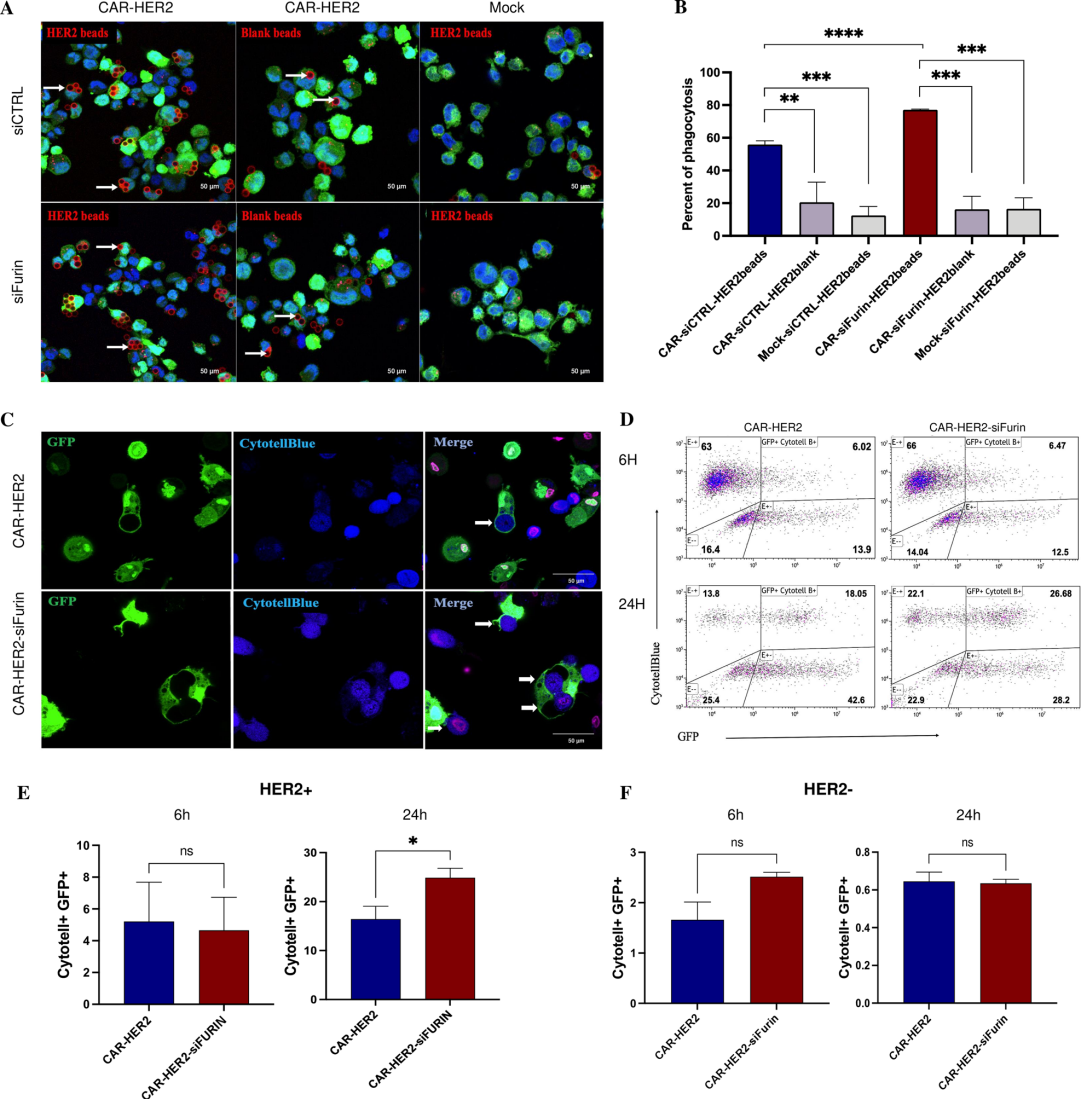
Furin inhibition enhances CAR-M-cell phagocytic activity. **A** Microscopy-based images of the phagocytosis of HER2-coated or control pH-Rodo-labeled beads by mock-transduced or THP-1-CAR cells, as observed by confocal microscopy. THP-1-CAR-M cells and mock-T cells were transfected with furin and control siRNAs. Nuclei (blue) were labeled with Hoechst, and beads (red) were stained with pHrodo. **B** Quantification of the internalization of HER2 beads and blank beads by the THP-1-CAR-M cells and mock-T cells (*n* = 3). **C** Immunofluorescence analysis of the phagocytosis of AU565 (HER2+) cancer cells by primary mock and CAR-M siControl or siFurin cells. The macrophages were GFP+, the cancer cells were CytotellBlue+, and the nuclei were stained with SYTOX^TM^ Deep Red Nuclei Acid Stain. **D** Representative FACS plots of phagocytosis after 6 and 24 hours of coculture at a 3:1 ratio (macrophage/cancer cells). The rate of phagocytosis was determined as the percentage of CytotellBlue+ and GFP+ cells among all cells. **E** Quantification of the phagocytosis of HER2+ cancer cells by mock and CAR-M cells after 6 and 24 hours of coculture (*n* = 3). **F** Quantification of the phagocytosis of HER2- cancer cells by mock and CAR-M cells after 6 and 24 hours of coculture (*n* = 3).

The next step was to evaluate the antitumor functions of the CAR-Ms in which furin was inhibited. For each experiment, siRNA transfection was conducted 24 hours before co-culture, as the decrease in furin expression was observed starting at 24 hours and persisted up to 72 hours post-transfection, encompassing the duration of the coculture (Supplementary Fig. 11). We first demonstrated the furin activity of CAR-Ms by the phagocytosis of HER2+ beads. CAR-Ms were transfected with either siRNA control (CAR-siCtrl) or siRNA furin (CAR-siFurin) and cocultured with HER2+ beads or blank beads. The percentage of phagocytosis was assessed as the number of CAR-Ms that phagocytized at least one bead. Approximately 55% of CAR-siCtrl and 80% of CAR-siFurin cells phagocytized at least one HER2+ bead (Fig. 4A, B). CAR-siFurin had significantly greater phagocytic activity against its target (Fig. 4B). Interestingly, this effect is specific for the HER2 antigen, as the phagocytic activity of the blank beads was much lower for both CAR-siCtrl and CAR-siFurin. Furthermore, Furin inhibition did not increase the phagocytic activity of macrophages that did not express the CAR receptor (WT, Fig. 4B). This effect is therefore specific to CAR-Ms. We also showed that CAR-siFurin increased HER2-specific phagocytosis of cancer cells/beads by immunofluorescence (Fig. 4C and Supplementary Fig. 12A, B). CAR-siFurin tended to phagocytose more than 2 beads per macrophage (Supplementary Fig. 12C). We measured the percentage of phagocytosis by flow cytometry as the percentage of double-positive CAR-M GFP+ and AU565 CytotellBlue+ cells. After 6 hours of coculture, no statistically significant difference was detected between the CAR-siCtrl group and the CAR-siFurin group. After 24 hours of coculture, the difference in phagocytosis between CAR-siCtrl and CAR-siFurin was statistically significant (Fig. 4E), with approximately 17% of CAR-siCtrl being double positive compared to 26% for CAR-siFurin (Fig. 4D), confirming the results obtained with the beads. No phagocytic activity was observed when CAR-M siFurin was co-cultured with HER2-negative cancer cells (Fig. 4F). In conclusion, we have shown that the tumor-killing capacity of CAR-Ms is significantly enhanced by furin inhibition.

### Furin silencing in CAR-Ms promotes proinflammatory activation

**Fig. 5:**
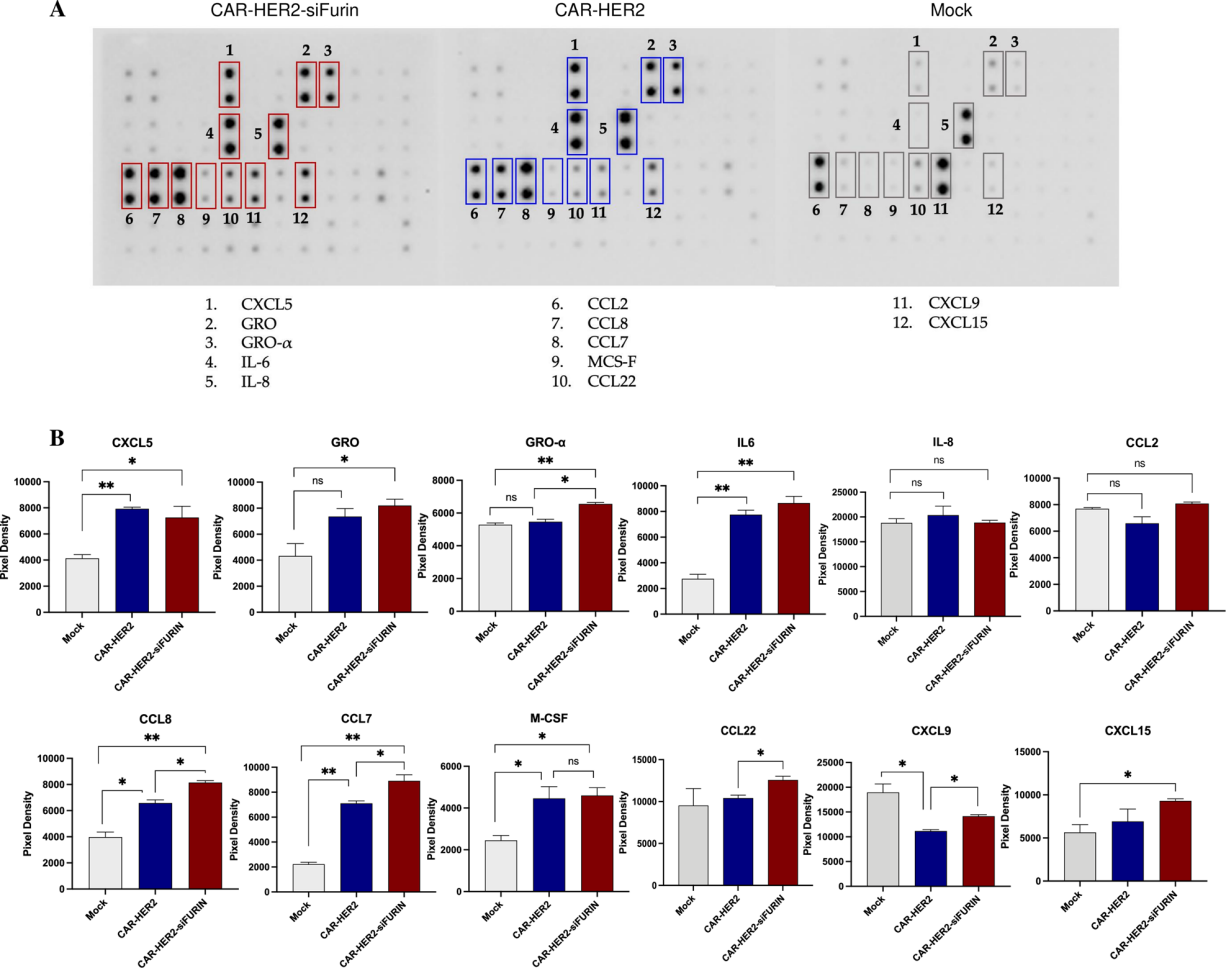
Inhibition of furin in CAR-Ms results in an increase in cytokine secretion. CAR-M-siFurin, CAR-M-siCTRL and mock control cells were cocultured with AU565 cells. After culturing for 24 hours, the collected secretome was added to a cytokine array. **A** Images depicting the cytokine arrays. **B** The intensities of the cytokine spots were measured using an iBright Imaging System and analyzed using ImageJ software, then represented on the graphs.

**Fig. 6:**
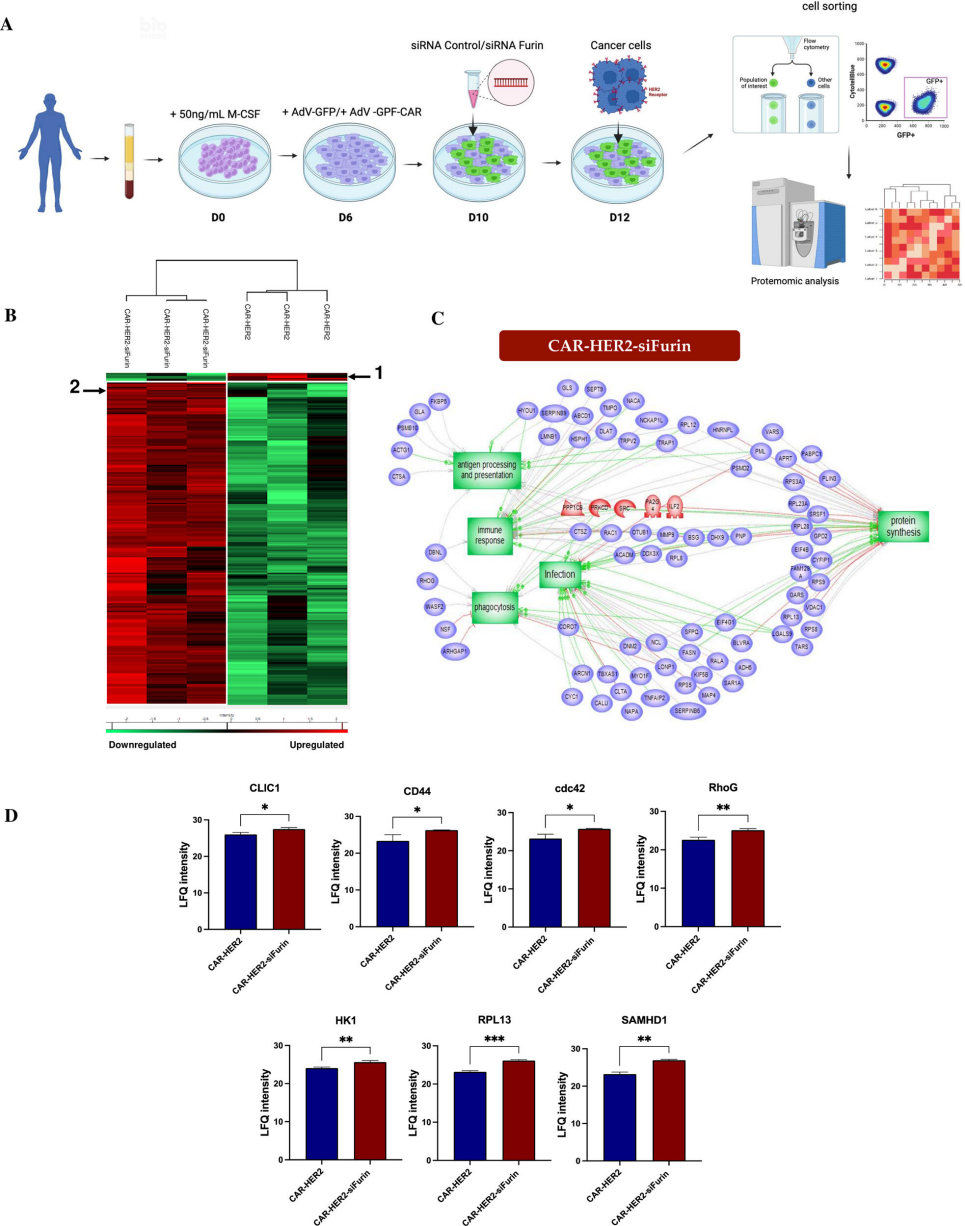
Furin inhibition maintains the proinflammatory phenotype of CAR-Ms in the presence of cancer cells. **A** Description of the procedure for mass spectrometry-based proteomics analysis of FACS-sorted CAR-Ms after coculture. CAR-Ms were transfected with furin siRNA (CAR-HER2-siFurin) or control siRNA (CAR-HER2). CAR-HER2 and CAR-HER2-siFurin were cocultured with the target cell line (AU565). After 24 hours of coculture, the cells were sorted by flow cytometry and lysed prior to analysis by FASP and LC‒MS/MS. **B** MaxQuant and Perseus software were used for the statistical analysis, and a heatmap was generated to show proteins that were significantly different between CAR-HER2 and CAR-HER2-siFurin. Two clusters of overexpressed proteins are highlighted (*n* = 3). **C** Global pathway analysis of proteins overexpressed in CAR-HER2-siFurin cells. **D** Label-free quantification of pro-inflammatory markers in CAR-HER2 and CAR-HER2-siFurin macrophages, with identification performed at a 0.01 FDR threshold.

A key feature of macrophages is their phenotypic plasticity to adapt to their local environment. Their phenotype can change in response to external signals. In tumors, the immunosuppressive environment can induce an anti-inflammatory macrophage phenotype. This can lead to a reduction in the efficacy of CAR-Ms. To determine whether furin inhibition can maintain the proinflammatory phenotype of CAR-Ms in the tumor microenvironment, we investigated the proinflammatory characteristics of macrophages after coculture with tumor cells. First, cytokine secretion levels were measured using a cytokine array (Fig. 5A). Among the cytokines tested, five exhibited significant differences in secretion between CAR-HER2 and CAR-HER2-siFurin: GRO-α (CXCL1), CCL8, CCL7, CCL22 and CXCL9. Furthermore, the secretion profiles of GRO (CXCL1, CXCL2 and CXCL3) and CXCL15 were significantly different between CAR-HER2-siFurin and mock macrophages (Fig. 5B). CCL8 and CCL7 were significantly more highly secreted by CAR-HER2 macrophages than by mock macrophages, and significantly more CCL8 and CCL7 were secreted by CAR-HER2-siFurin-treated macrophages than by CAR-HER2 macrophages. GRO chemokines were significantly secreted by CAR-HER2-siFurin, and among these, significantly more GRO-α was secreted by CAR-HER2-siFurin than by CAR-HER2. CCL22, CXCL9, and CXCL15 were significantly more highly secreted by CAR-HER2-siFurin than by CAR-HER2. All these factors have a chemoattractant role in the recruitment of other immune cells, and some of them, such as CXCL9, promote immune cell proliferation [27]. We then investigated the effect of furin inhibition on the CAR-M proteome after coculture. We transduced macrophages with the CAR construct and transfected them with siRNA control or siRNA furin 4 days later. Two days after transfection, we added target cancer cells to the macrophages and maintained the coculture for 24 hours before FACS sorting of the CAR-Ms based on GFP expression (Fig. 6A). The sorted CAR-Ms were then lysed, and proteins were extracted before trypsin digestion and analysis by liquid chromatography coupled with mass spectrometry (LC‒MS). A total of 467 proteins were identified (Supplementary Table 4). To identify proteins with the most prominent differences in expression profiles between CAR-siFurin and CAR-siCtrl, we used Student’s t test with a false discovery rate (FDR) of 5%. A total of 193 differentially expressed proteins were identified (Supplementary Table 5). The significantly up- and downregulated proteins between the two conditions are shown on a heatmap (Fig. 6B). An enrichment analysis was performed for the cluster 2 proteins indicated in the heatmap, corresponding to proteins overexpressed in CAR-siFurin macrophages. The main enriched biological pathways were related to antigen processing and presentation, immune response, infection, phagocytosis and protein synthesis (Fig. 6C). As examples of proteins involved in antigen presentation, PSMB10, a member of the immunoproteasome; ACTG1, an actin remodeling protein; and PML, a protein known to induce the expression of genes related to MHC class I antigen presentation [28], were overexpressed in CAR-M siFurin. As examples of proteins involved in phagocytosis, CTSZ, a lysosomal cysteine protease, and Rac1, a member of the Rho family of GTPases, were overexpressed in CAR-M siFurin. Interestingly, several activation markers were upregulated in furin-inhibited CAR-M after co-culture with cancer cells (Fig. 6D). These markers include phagocytic proteins such as CLIC1, known to be induced in activated macrophages [29]; CD44, a receptor critical for phagocytosis [30]; Cdc42, which regulates the actin rearrangement required for phagocytosis [31]; and RhoG, a Rho GTPase that supports phagocytic activity [32]. Additional proteins involved in the pro-inflammatory activation of macrophages were also upregulated in furin-inhibited CAR-M, such as HK1, which promotes inflammatory cytokine production [33]; RPL13, involved in NFκB and IFN-β pathway activation [34]; and SAMHD1, a protein induced by pro-inflammatory signals and necessary for IFN-γ-dependent activation [35]. CD14 expression, a co-receptor for LPS, was elevated in furin-inhibited CAR-M compared to control CAR-M, consistent with our previous findings (Supp. Fig. 13). Taken together, these results demonstrate that Furin inhibition not only promotes the phagocytic activity of CAR-Ms but also maintains a proinflammatory phenotype in contact with cancer cells, which is critical for maintaining therapeutic efficacy over time. The increased expression of antigen-presenting proteins and secretion of chemokines by CAR-Ms may also promote T-cell recruitment and activation to enhance antitumor activity.

### Depletion of Furin in CAR-Ms reduces the growth of breast cancer tumoroids

**Fig. 7:**
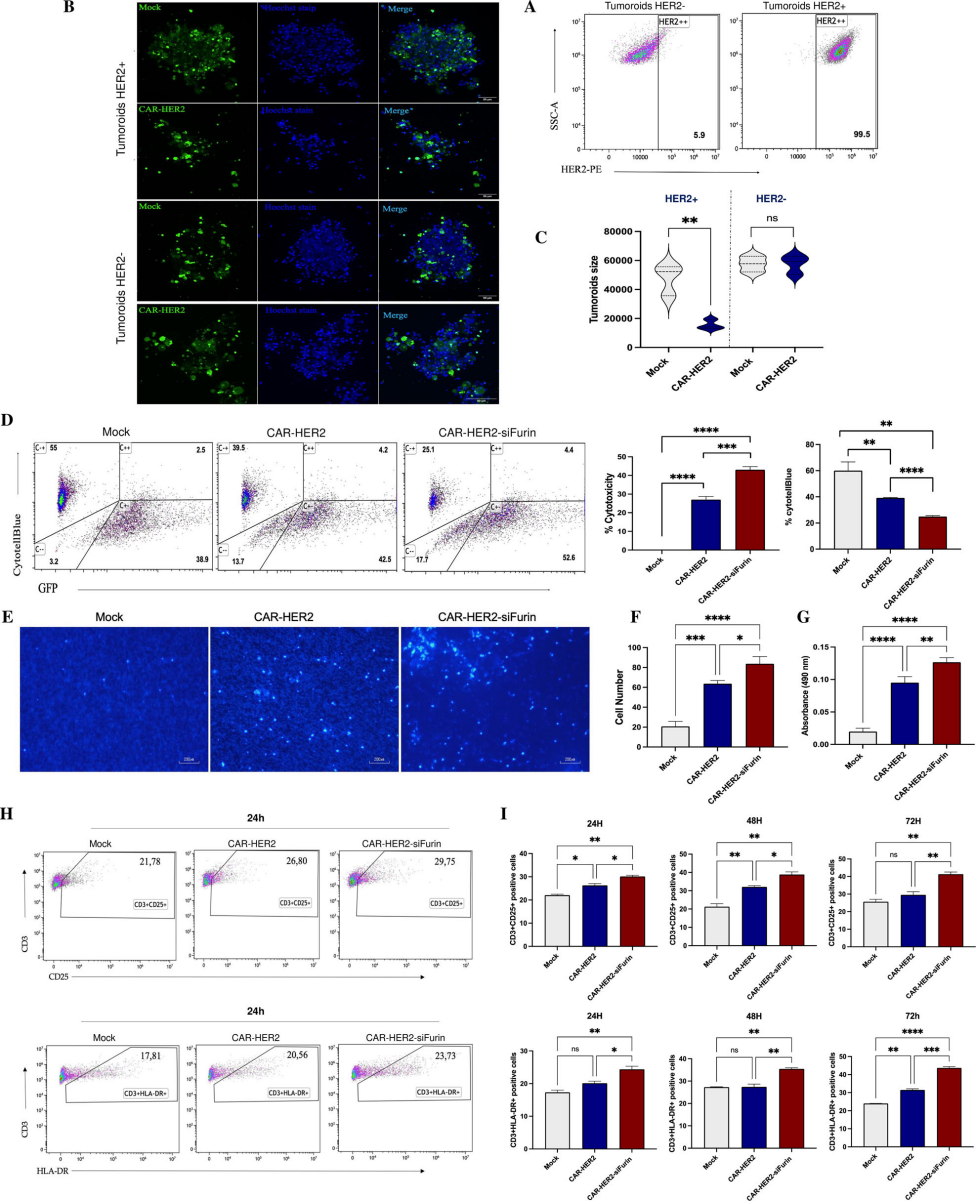
Furin inhibition in CAR-Ms reduces the viability of HER2+ breast cancer patient tumoroids and activate T cells. **A** FACS plots showing the percentage of primary breast cancer cells derived from HER2- and HER2+ tumoroids that express the HER2 antigen. **B** Immunofluorescence analysis of the cytotoxic effect of CAR-Ms on tumoroids (HER2+ and HER2-) in comparison with that of mock macrophages. The macrophages are GFP + , and the nuclei (blue) are labeled with Hoechst. **C** Quantification of the size of HER2+ and HER2- tumoroids in the presence of CAR-Ms or mock controls using ImageJ software (*n* = 3). **D** FACS plots showing the percentage of phagocytosis of primary breast cancer cells by mock, CAR-HER2, and CAR-HER2-siFurin macrophages. The graph shows the quantification of the cytotoxicity and the remaining cancer cells after coculture (*n* = 3). **E** Number of T cells that migrated under each condition: Mock, CAR-HER2, and CAR-HER2-siFurin conditioned medium, evaluated using microscopy. **F** Quantification of T cells that adhered to the transwell membrane. **G** Quantification of T cells that migrated to the bottom of the well under the specified conditions, measured using an MTS cell proliferation assay. **H** Scatter plots illustrating the frequency of CD25+ and HLA-DR+ lymphocytes. **I** The graphs represent the ratio of CD25+ and HLA-DR+ lymphocytes in CAR-HER2 and CAR-HER2-siFurin conditions relative to mock conditions after 24, 48, and 72 h of coculture (*n* = 2).

We then sought to evaluate the antitumor activity of furin-inhibited CAR-Ms in a more complex tumor model. We established 3D tumoroids made from human primary breast cancer biopsies, as we have previously published [36]. Tumoroids made from two different breast cancer subtypes were used to test the efficacy of CAR-M. We first checked the HER2 expression status of the tumoroids before they started coculture using flow cytometry. More than 99% of the cells in the HER2+ tumoroids expressed the HER2 protein, while only 5.9% of the cells in the HER2- tumoroids expressed it (Fig. 7A). We then established a coculture system of tumoroids with CAR-Ms in a nonadhesive plate in complete tumoroid medium supplemented with 2% Matrigel. Prior to coculture, macrophages were transduced with the CAR or the mock construct, and 4 days later, they were harvested and cocultured with the tumoroids at a ratio of 3 macrophages to 1 tumor cell for 48 hours. Light sheet microscopy was then performed on cleared tumoroids to assess macrophage infiltration into the whole tumoroids. Representative images are shown in Fig. 7B, where we can clearly see CAR-Ms and mock macrophage infiltration in HER2+ and HER2- tumoroids. Interestingly, the HER2+ tumoroids appeared disorganized in the presence of CAR-Ms compared to those in the presence of mock macrophages. We confirmed these observations by quantifying the tumoroid size under all conditions (Fig. 7C). A reduction in the size of HER2+ tumoroids was observed only when they were cocultured with CAR-Ms. This was not the case for HER- tumoroids. We next investigated the effect of furin silencing on CAR-M-mediated phagocytosis in a tumoroid model. After the macrophages were transduced as described above, they were transfected with either the siRNA control or the siRNA furin. One day later, the cells were harvested and cocultured with the tumoroids for 48 hours at a ratio of 3 macrophages to 1 tumor cell. CAR-M activity was assessed by FACS (Fig. 7D). The percentage of cytotoxicity for CAR-M siFurin was 40%, whereas it was 27% for CAR-M siControl (Fig. 7D). This increased cytotoxicity of CAR-M siFurin was correlated with the reduced number of tumor cells stained with Cytotell Blue after coculture (Fig. 7D; 25% of residual tumor cells for CAR-M siFurin compared to 43% for CAR-M siControl). Finally, we wanted to evaluate the ability of CAR-M siFurin to activate other immune cells, such as T lymphocytes, to induce a synergistic effect. A chemoattraction assay, described in Supplementary Fig. 14, demonstrated that factors secreted by furin-inhibited CAR-M in co-culture with tumor cells attracted more T lymphocytes (Fig. 7E). We measured both the number of T cells adhered to the transwell membrane (Fig. 7F) and those that migrated to the bottom of the well (Fig. 7G). Next, T lymphocytes were cultured for 48 hours with conditioned medium from CAR-Ms and tumoroid cocultures, their proliferation was assessed (Supplementary Fig. 15A). As a positive control, T lymphocytes were cultured with CD3/CD28 beads, and as a negative control, T lymphocytes were cultured in RPMI medium alone. We confirmed that factors secreted by CAR-M siFurin induced greater proliferation of T lymphocytes than those secreted by CAR-M siControl or mock macrophages (Supplementary Fig. 15B). Finally, when T cells were co-cultured with siFurin-inhibited CAR-M-tumoroids, they exhibited increased activation, as indicated by higher expression of early and late activation markers, CD25 and HLA-DR respectively (Fig. 7H, I, Supplementary Fig. 16). We confirmed this through a global proteomics analysis, identifying several T-cell activation markers when T cells were co-cultured with furin-inhibited CAR-M-tumoroids. Examples of markers overexpressed in T-cells under this condition include the costimulatory molecule CD81, the activation markers CD82 and CD69, the cytotoxic proteases Granzyme A and Granzyme B and the pro-inflammatory cytokine IL-16 (Supplementary Fig. 17, Supplementary Tables 6 and 7).

Taken together, these results confirmed in a complex model that furin-inhibited CAR-M phagocyte tumoroids more efficiently than control CAR-M and have the potential to activate T cells.

## Discussion

Previous studies have shown that the proprotein convertases furin and PC1/3 play critical roles in controlling macrophage polarization in mouse and rat macrophages [11–14, 23, 26]. In this study, we investigated the role of these two enzymes in the proinflammatory polarization of human macrophages. Two models were studied: THP-1-derived macrophages and primary blood monocyte-derived macrophages. While both enzymes were expressed by THP-1 macrophages, only furin was expressed by primary macrophages. PC1/3 was originally thought to be expressed exclusively in the neuroendocrine system [8], but studies have shown that it is also expressed in immune organs [37]. PC1/3 expression and trafficking can be modulated by TLR ligands such as CpG-ODN [13] and LPS [23] in mouse and rat macrophages. Thus, we can expect that PC1/3 expression is also induced by TLR ligands in human macrophages, but as our aim is to develop a therapeutic strategy, we wanted to study both the basal expression of PC enzymes and the tumor context. In contrast, furin has a more ubiquitous expression pattern [8]. Its expression has been detected in the basal state in human macrophages, and we have shown for the first time that the coculture of macrophages with cancer cells results in increased furin expression in human macrophages. Furin is known to have an anti-inflammatory role, promoting Arg1 expression in mouse macrophages and the production of the anti-inflammatory cytokine TGF-β1 [11]. Furin is also involved in the regulation of the T-cell phenotype, as its deletion in T cells results in an overproduction of cytokines and autoantibodies and in the development of inflammatory diseases [10, 38]. Therefore, we believe that cancer cells hijack the antitumor activity of macrophages by inducing anti-inflammatory furin expression, which may be a new escape strategy for cancer cells. Indeed, we have shown that furin inhibition in THP-1 macrophages and primary macrophages triggers their proinflammatory activation. In addition, furin inhibition appears to increase the number of functional macrophages, as evidenced by increased expression of proteins involved in phagocytosis and increased expression of costimulatory molecules required for antigen presentation, such as CD86. Furin inhibition may therefore be used as a therapeutic strategy to reactivate antitumor immunity. In fact, several studies have demonstrated the potential use of a PC inhibitor as an antitumor immunotherapy [15, 17–19]. However, an effective immunotherapeutic strategy cannot be based on furin inhibition alone. Furin inhibition perpetuates a proinflammatory state and may therefore be of interest in combination with other immunotherapeutic strategies aimed at restoring the antitumor function of immune cells, such as CAR therapies. CAR-T-cell therapies have revolutionized the field of cellular immunotherapy for the treatment of hematological cancers [1]. Many challenges limit their efficacy in solid tumors. CAR-Ms have emerged to overcome these limitations, as they comprise the majority of immune cells that infiltrate the tumor environment. A first clinical trial in patients recently started to demonstrate the efficacy of CAR-Ms in detecting and eliminating solid tumors [5, 39] (CT-0508 phase 1 first-in-human study). These studies demonstrate that phagocytic activity is effectively induced by a full CAR receptor and not by an scFv alone, as a CAR lacking an intracellular signaling domain does not drive phagocytosis in macrophages [40]. The challenge is to develop second-generation CAR-Ms that are even more effective because once macrophages infiltrate a tumor, they can differentiate into anti-inflammatory macrophages and lose their antitumor function. In this context, inhibiting furin in CAR-Ms is a way to enhance their antitumor activity. Our findings revealed that furin inhibition in CAR-Ms not only promotes the phagocytic activity of CAR-Ms against breast cancer cells but also maintains a pro-inflammatory phenotype in contact with cancer cells, which is critical for maintaining therapeutic efficacy over time. We were also able to demonstrate greater phagocytic activity against tumoroids derived from the tumors of breast cancer patients. In addition, by eliminating tumor cells, CAR-Ms may open the door in the solid TME to other immune cells, including T lymphocytes. This collaborative strategy could be the key to making immunotherapy a novel impactful strategy for the treatment of solid tumors. We have herein shown here that CAR-Ms can activate T-cells, and that furin inhibition amplifies this phenomenon.

In conclusion, our results showed that we can generate a second generation of CAR-Ms with durable proinflammatory and antitumor phenotypes, which represents a promising approach for CAR-based immune cell therapy.

## Supplementary information

supplementary figures

Original data

List of proteins overexpressed in the cluster highlighted in Supplementary Fig. 17.

Total matrix extracted from the Perseus file containing the list of proteins identified in T lymphocytes following co-culture with CAR-M-tumoroids.

Total matrix extracted from the Perseus file containing the list of proteins identified in THP-1 macrophages after siRNA-mediated inhibition of furin and PC1/3

List of overexpressed proteins in Clusters 1, 2, 3, 4 and 5 from the heatmap shown in Figure 2

List of exclusive proteins expressed in THP-1 macrophages after siRNA-mediated inhibition of furin and PC1/3 (with reference to Figure 3)

Total matrix extracted from the Perseus file containing the list of proteins identified in CAR-Ms after siRNA-mediated inhibition of furin

List of overexpressed proteins in clusters 1 and 2 from the heatmap shown in Figure 7

## Data Availability

The data are available upon request. All data relevant to the study are included in the article or uploaded as supplementary information.
