# Supplementary material for: Inhibition of furin in CAR macrophages directs them toward a proinflammatory phenotype and enhances their antitumor activities
Source: Cell Death Dis. 2024 Dec 4;15(12):879. doi: 10.1038/s41419-024-07267-4 (PMC11618602; doi:10.1038/s41419-024-07267-4)
Supplement: Supplementary file 1 — supplementary figures [file 41419_2024_7267_MOESM1_ESM.docx]

**Inhibition of furin in CAR macrophages directs them towards a pro-inflammatory phenotype and enhances their anti-tumor activities.**

Lydia Ziane Chaouche^1^, Antonella Raffo-Romero^1^, Nawale Hajjaji^1,2^, Firas Kobeissy^3^, Donna Pinheiro^1^, Soulaimane Aboulouard^1,^ Adeline Cozzani^4^, Suman Mitra^4^, Isabelle Fournier^1^, Dasa Cizkova^1,5,6^, Michel Salzet^1^*, Marie Duhamel^1^*

**Supplementary information file**


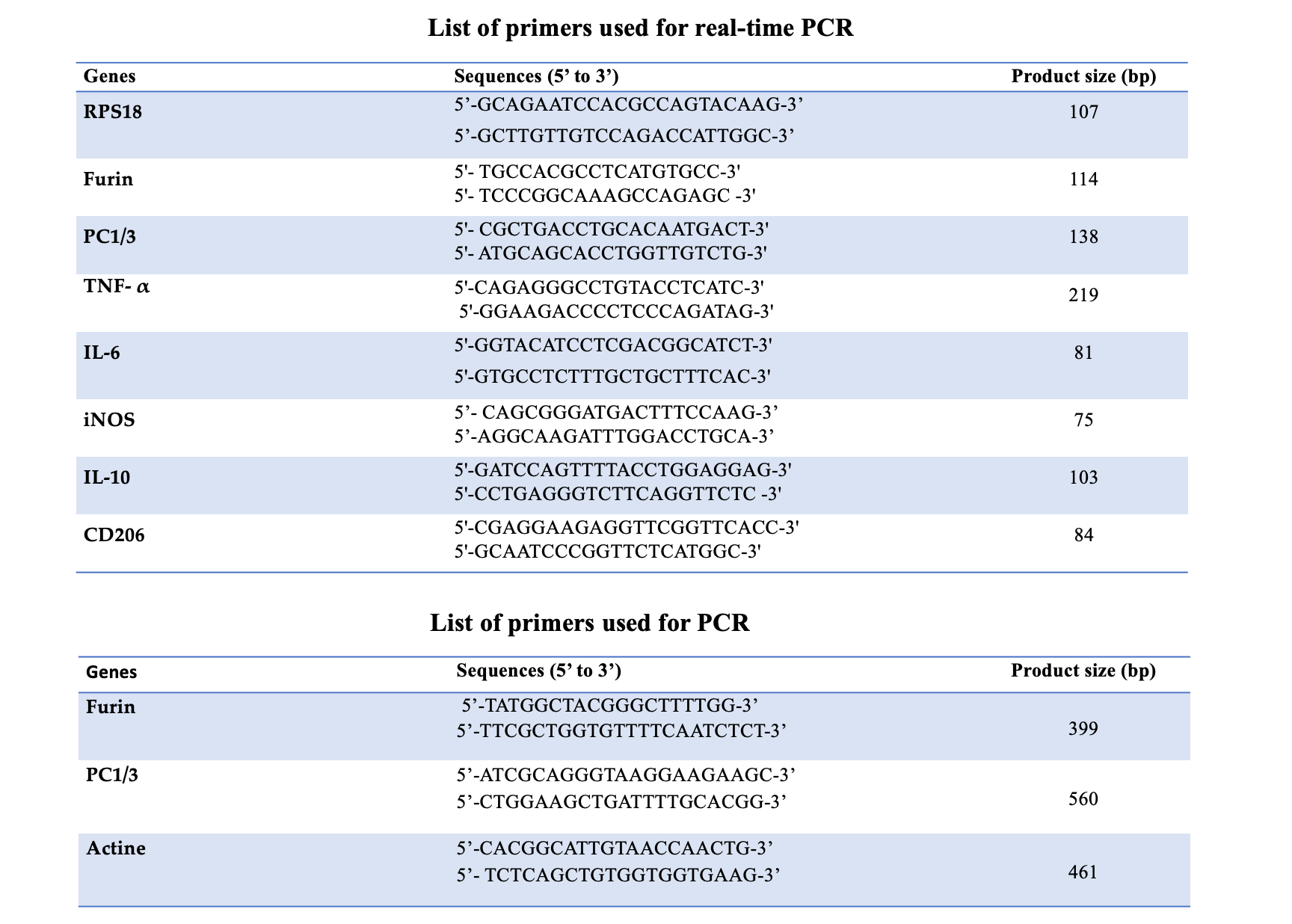


**Supp. Figure 1**: List of primers used for real-time PCR and PCR.


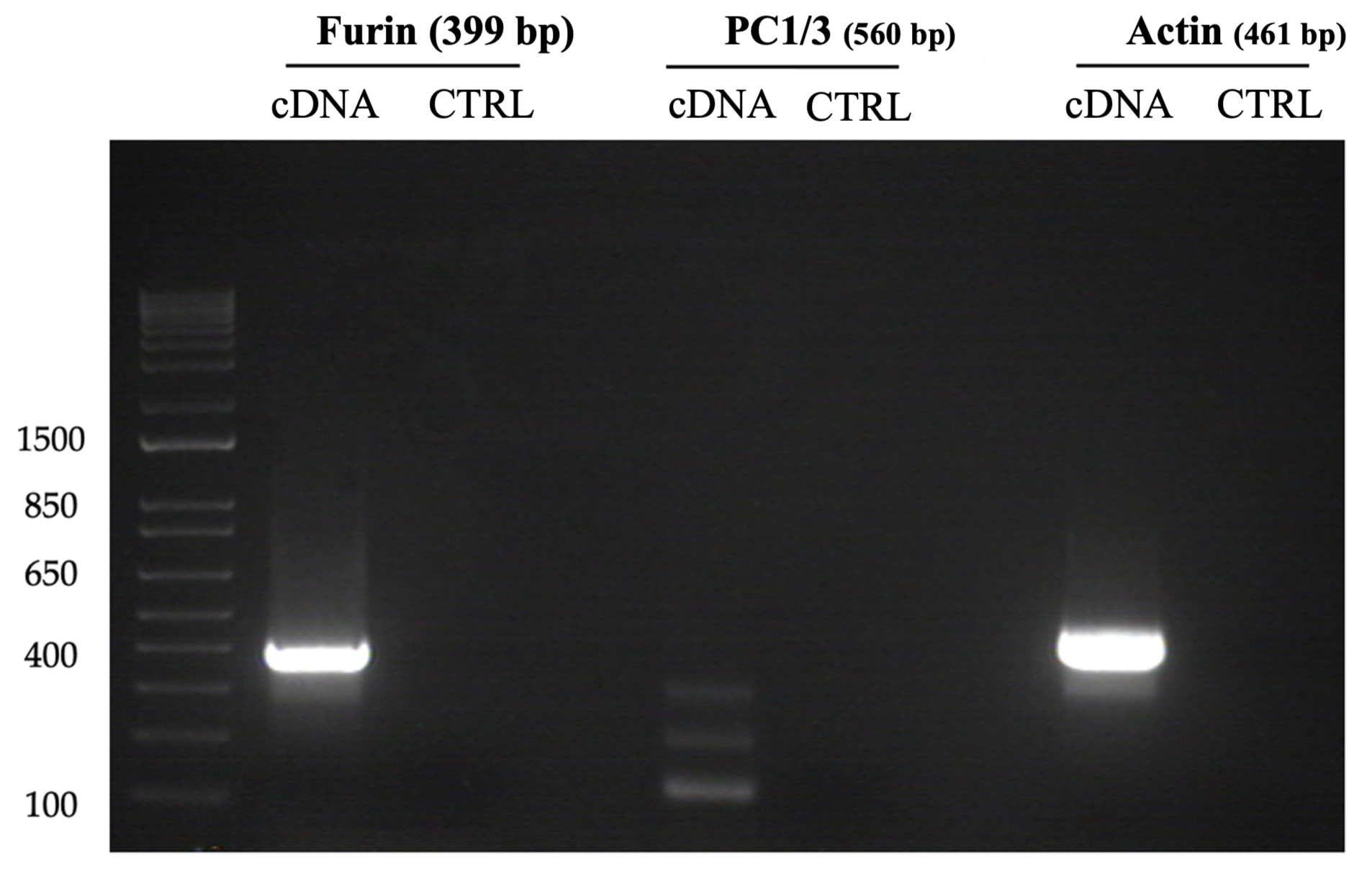


**Supp. Figure 2:** **RT-PCR amplification of Furin and PC1/3 in primary macrophages**. Agarose gel electrophoresis showing PCR products. Lane 1: DNA marker. Lanes 2-7: PCR products of furin (lane 2), PC1/3 (lane 4) and actin (lane 5). In lanes 2, 4 and 6, complementary DNA (cDNA) was added, representing experimental conditions. Lanes 3, 5 and 7 serve as negative controls (CTRL), where no cDNA was added.


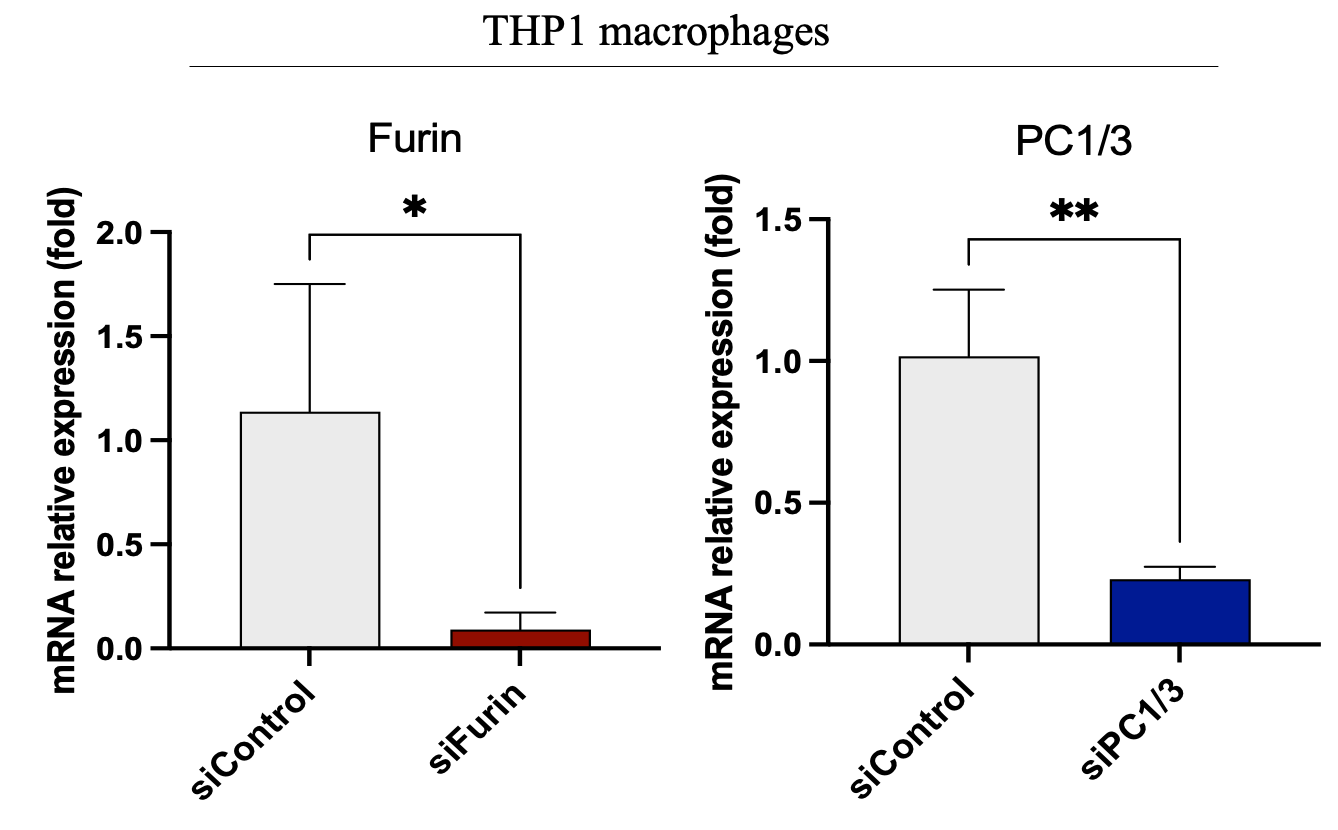


**Supp. Figure 3:** Inhibition of Furin and PC1/3 in THP1 macrophages. Relative mRNA levels of PC1/3 and furin after siRNA inhibition in THP-1 macrophages. The fold change is expressed relative to the mRNA levels obtained from cells treated with the siRNA control (n=3).


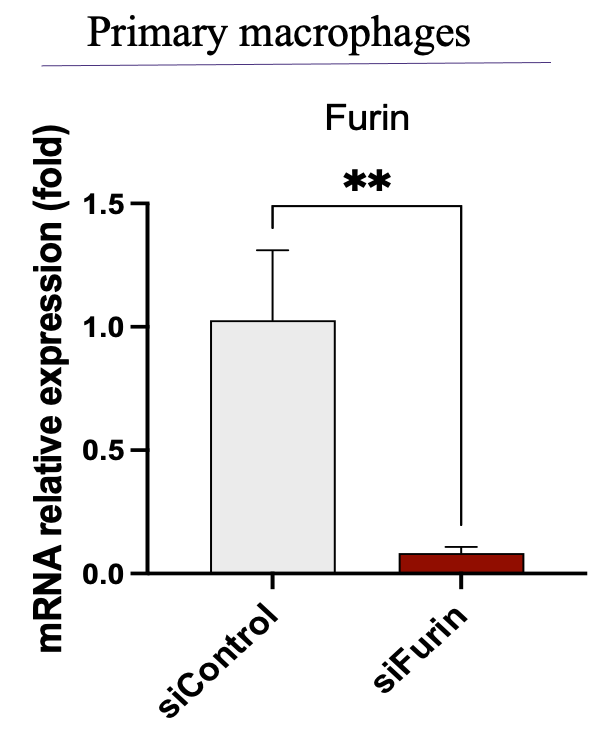


**Supp. Figure 4**: Inhibition of Furin in primary macrophages. Relative mRNA levels of furin after siRNA inhibition in primary macrophages. The fold change is expressed relative to the mRNA levels obtained from cells treated with the siRNA control (n=3).


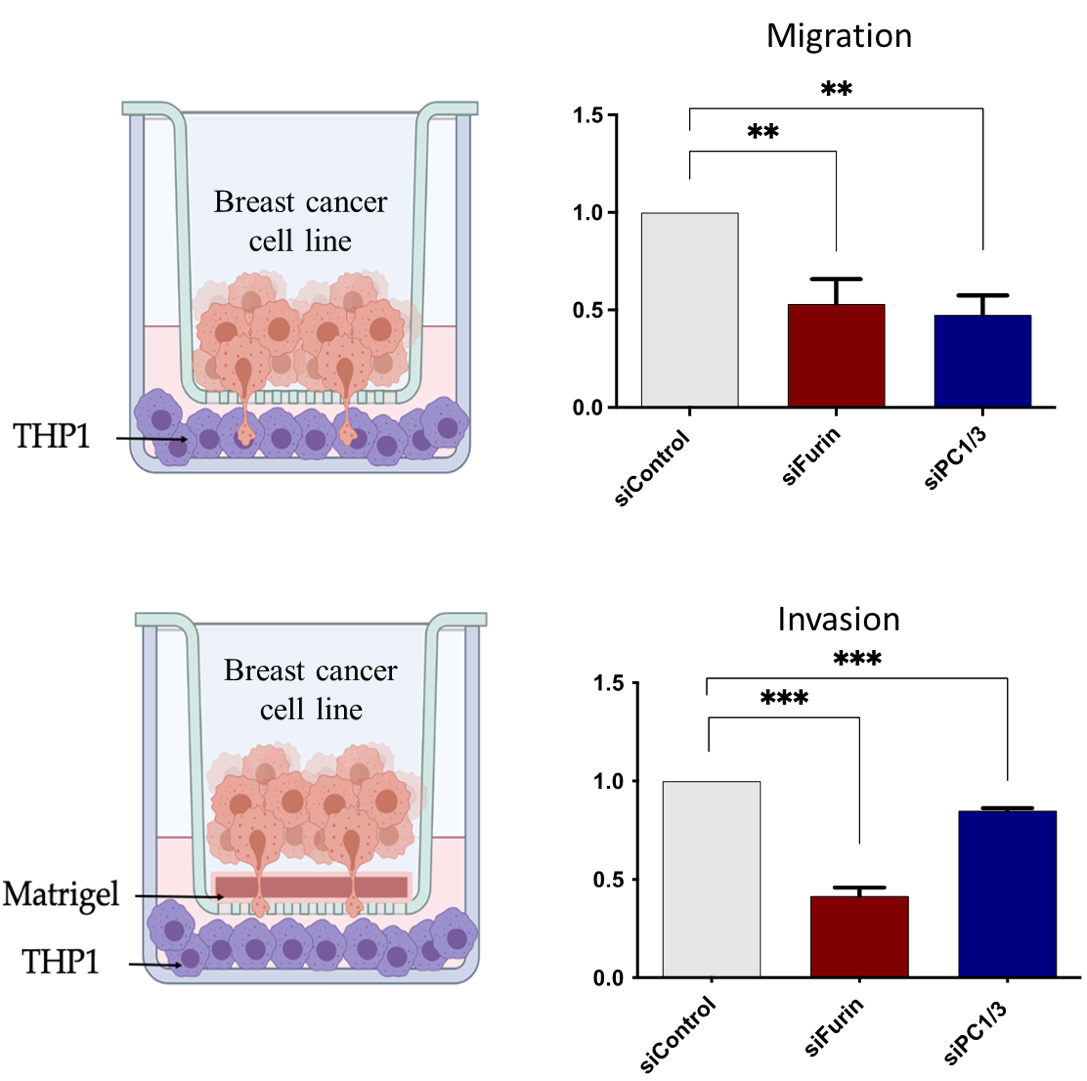


**Supp. Figure 5:** **Inhibition of Furin and PC1/3 reduces cancer cell migration and invasion**. The Transwell assay was used to determine cancer cell migration and invasion. The number of cancer cells migrating and invading in the presence of THP1 transfected with siFurin, siPC1/3 and siCTRL is shown in the statistical graphs (n=3).


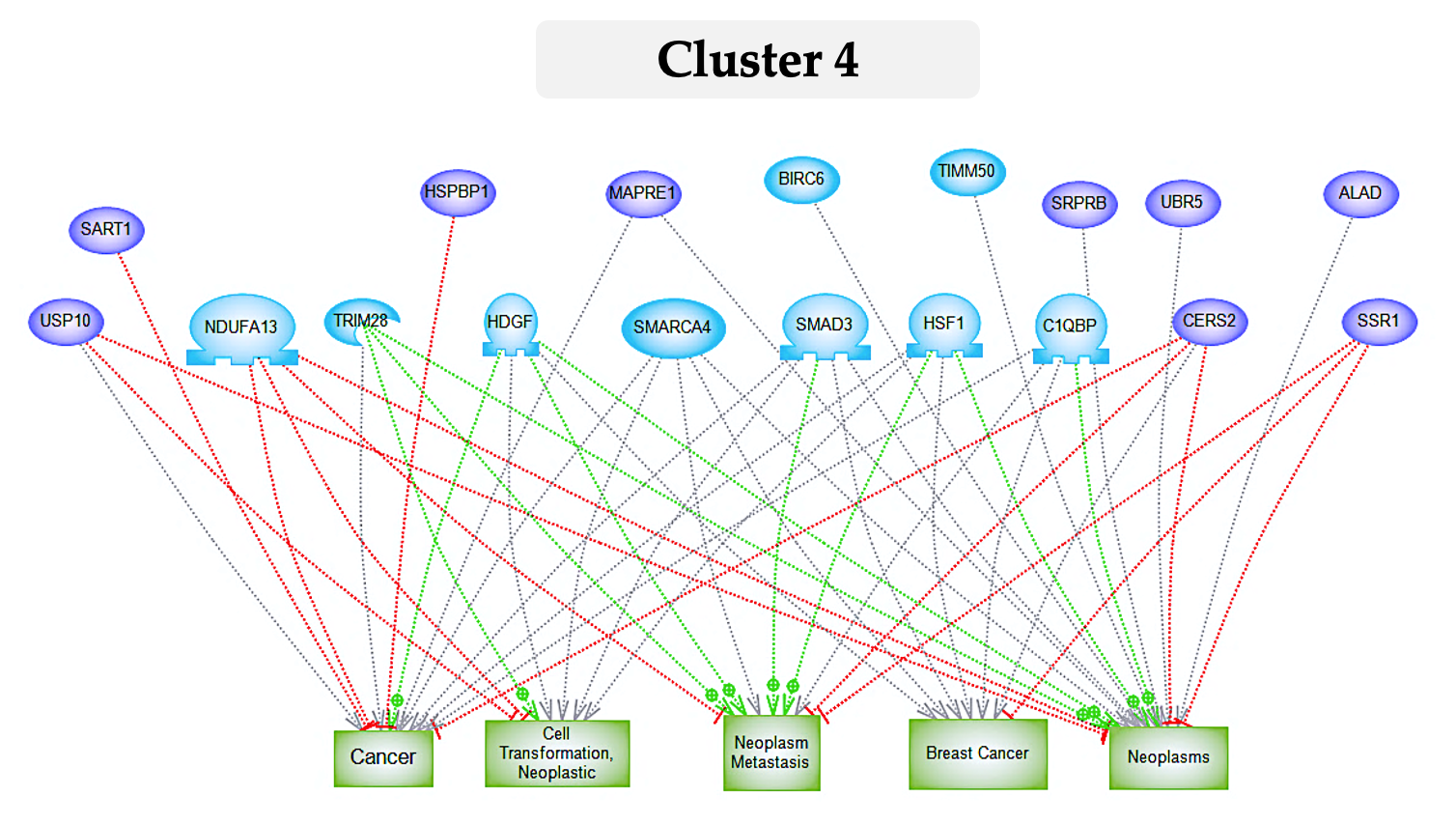


**Supp. Figure 6: Global analysis of protein pathways expressed in THP1 WT**. Cluster 4 represents overexpressed proteins in THP1 WT cells.


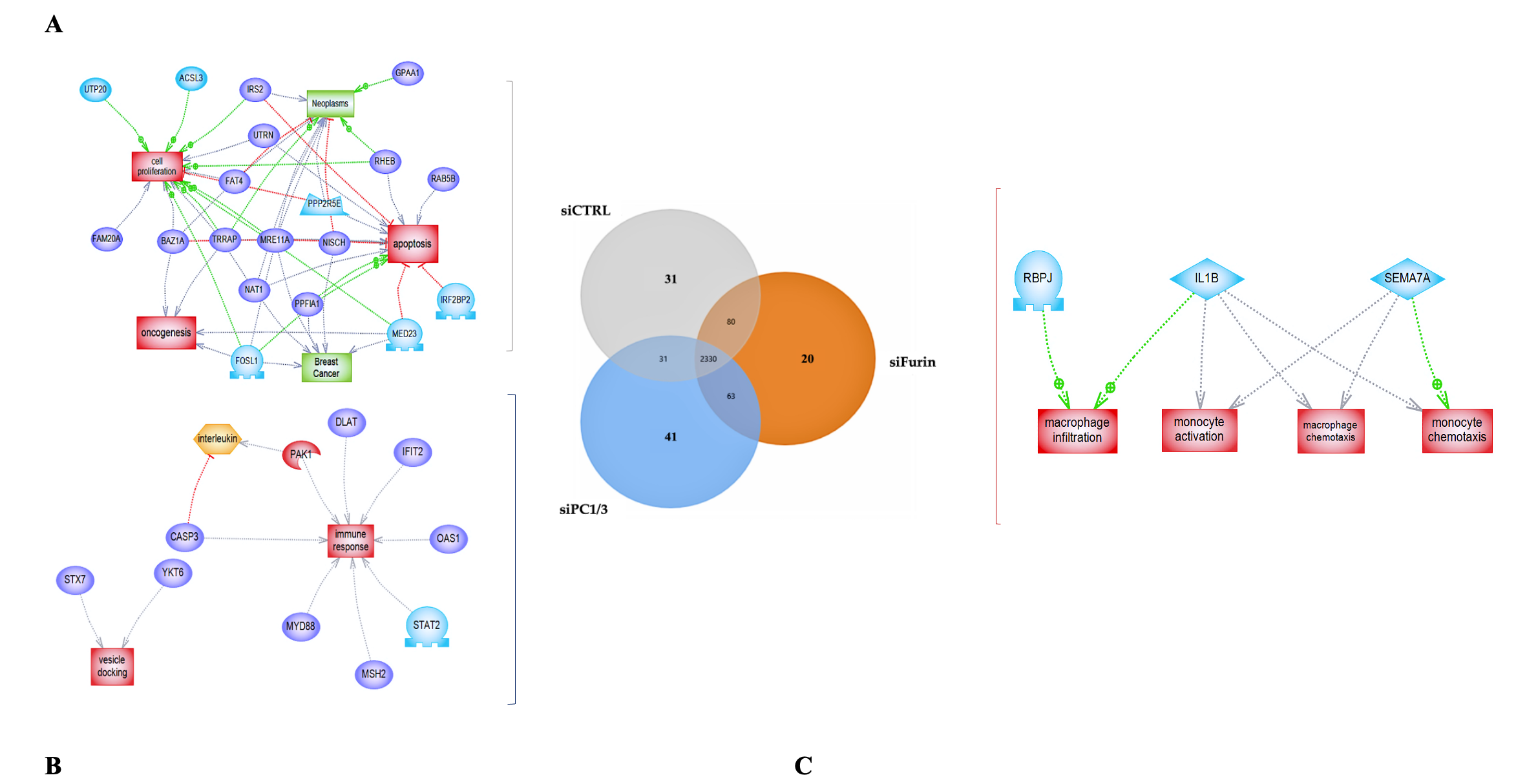


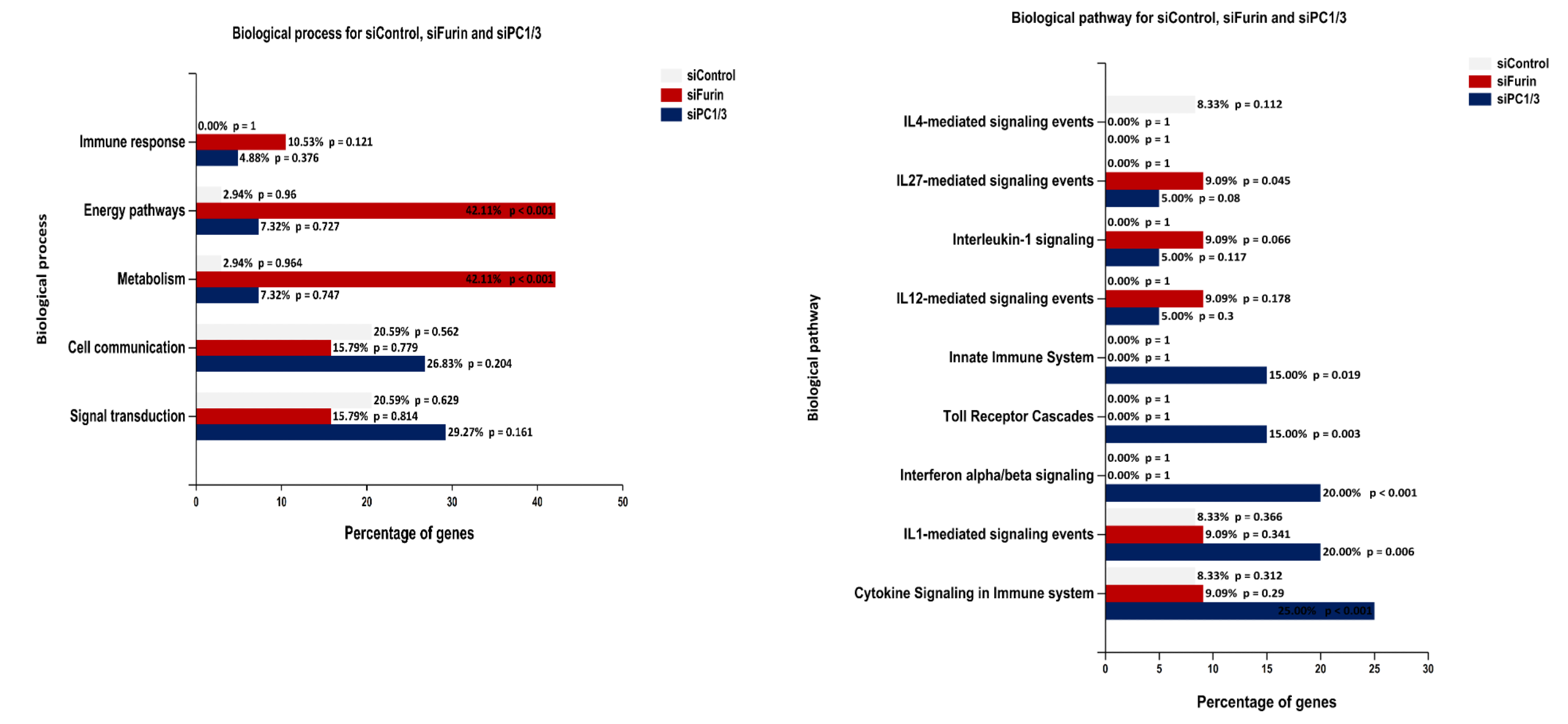


**Supp. Figure 7: Exclusive proteins expressed in Furin- and PC1/3-inhibited THP1 macrophages are associated with the pro-inflammatory response**. A) Venn diagram showing exclusive proteins in each condition (siFurin, siPC1/3 and siControl) and global analysis of exclusive protein pathways expressed in THP1 transfected with different siRNAs (siFurin, siPC1/3 or siCtrl). B-C) Biological processes of exclusive proteins identified in THP1 siControl, siFurin and siPC1/3. Analysis was performed using FunRich software.


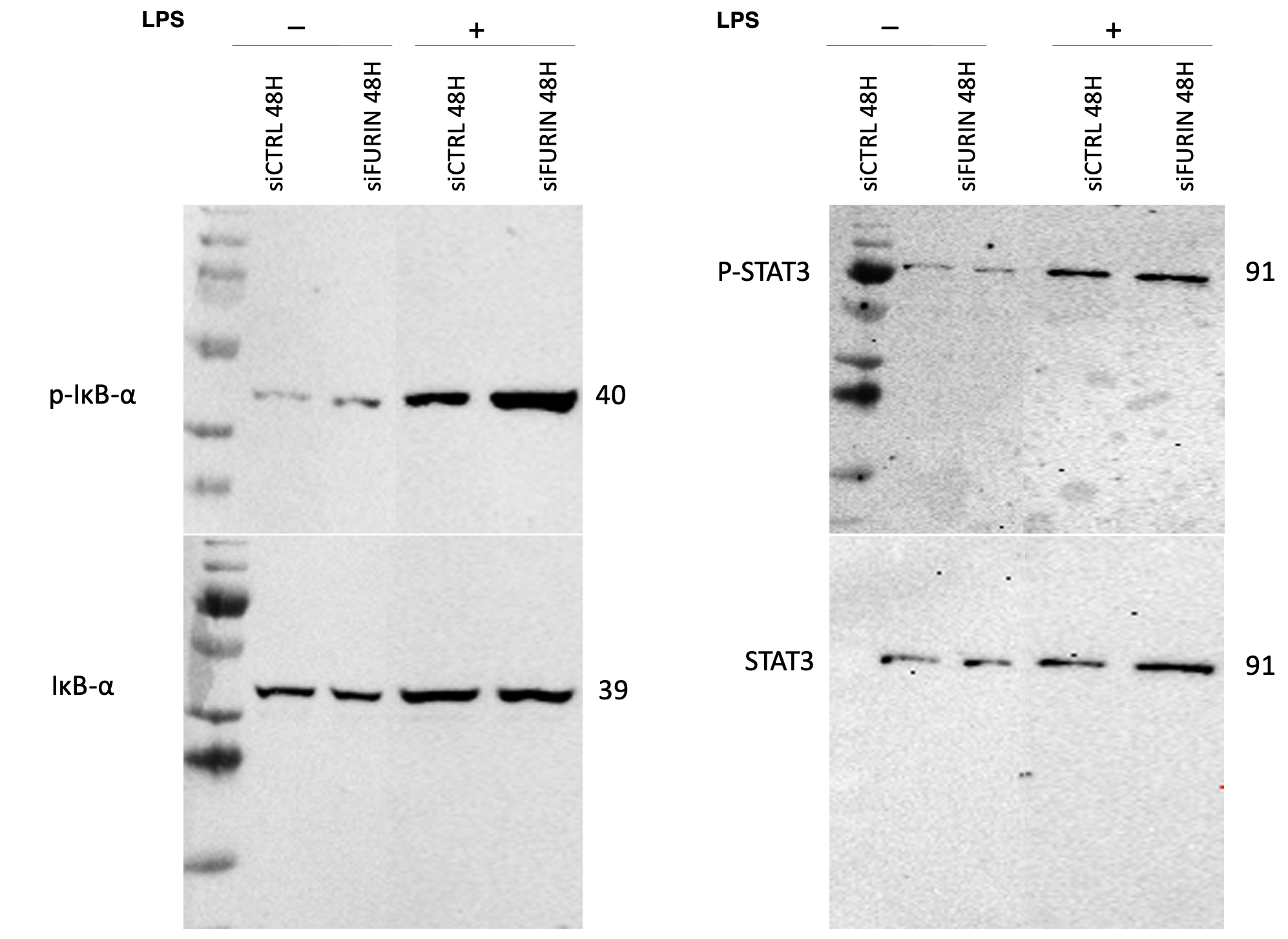


**Supp. Figure 8: Raw images of Western blot membranes.** The analysis shows the levels of phospho-IκB-α, total IκB-α, phospho-STAT3, and STAT3 in primary macrophages transfected with furin or control siRNAs, with or without LPS stimulation for 3 hours (200 ng/mL).


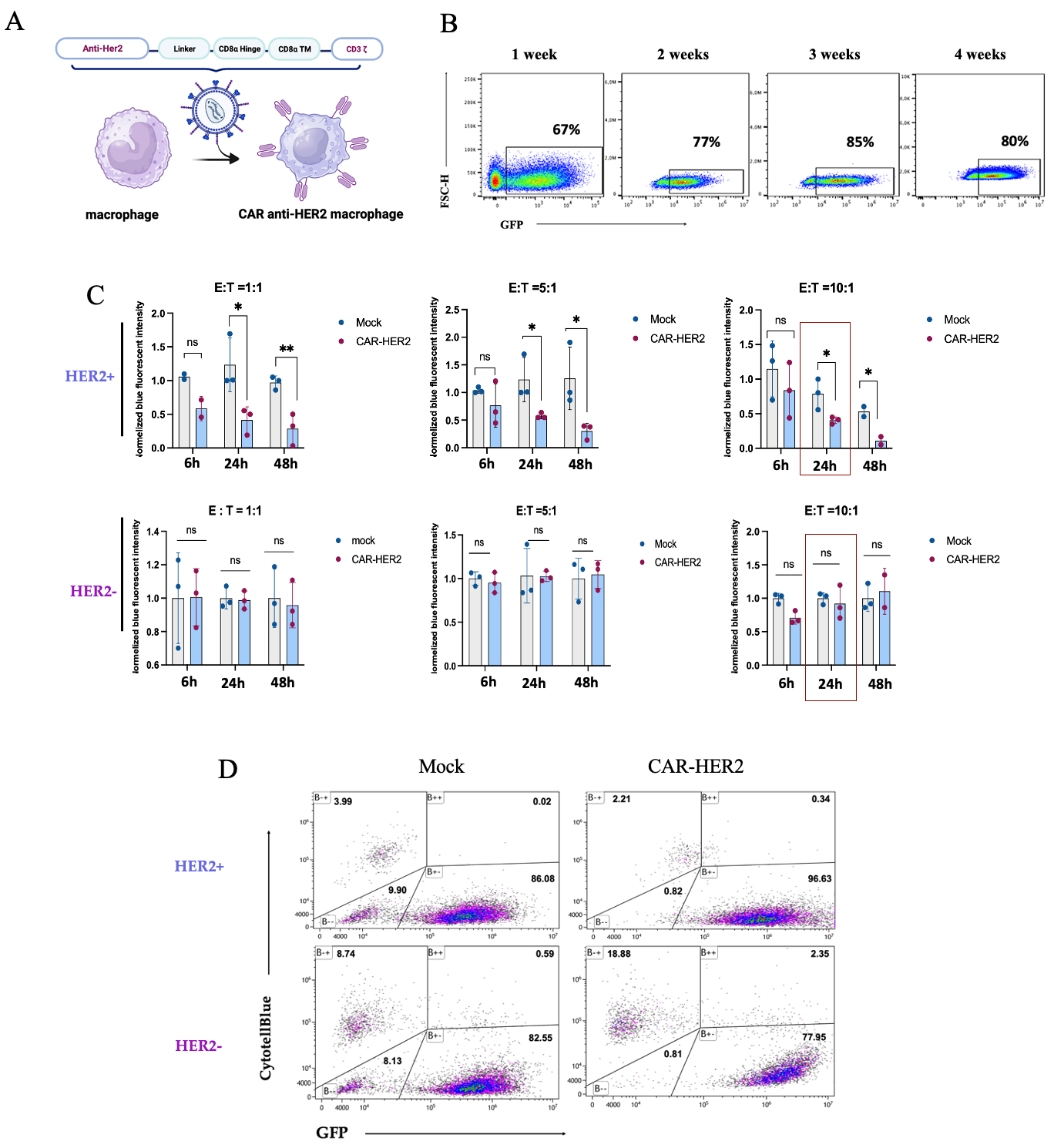


**Supp. Figure 9: Targeted anti-tumor activity of THP1-CAR macrophages**. A) Illustration of the chimeric antigen receptor sequence. B) Analysis of the stability of GFP protein expression over time. THP1 were analyzed by flow cytometry 1, 2, 3 or 4 weeks after transduction. C) Quantification of remaining HER2+ and HER2- cancer cells after coculturing with CAR or mock macrophages at different effector: target ratios (1:1, 5:1 and 10:1) for 6h-24h-48h, analysed by flow cytometry. Phagocytosis rate is determined as the % of BFP+ cells out of all cells (n=3). D) Representative FACS plots of phagocytosis after 24 hours of coculture with a 10:1 macrophage/HER2+ or HER2- cancer cell ratio.


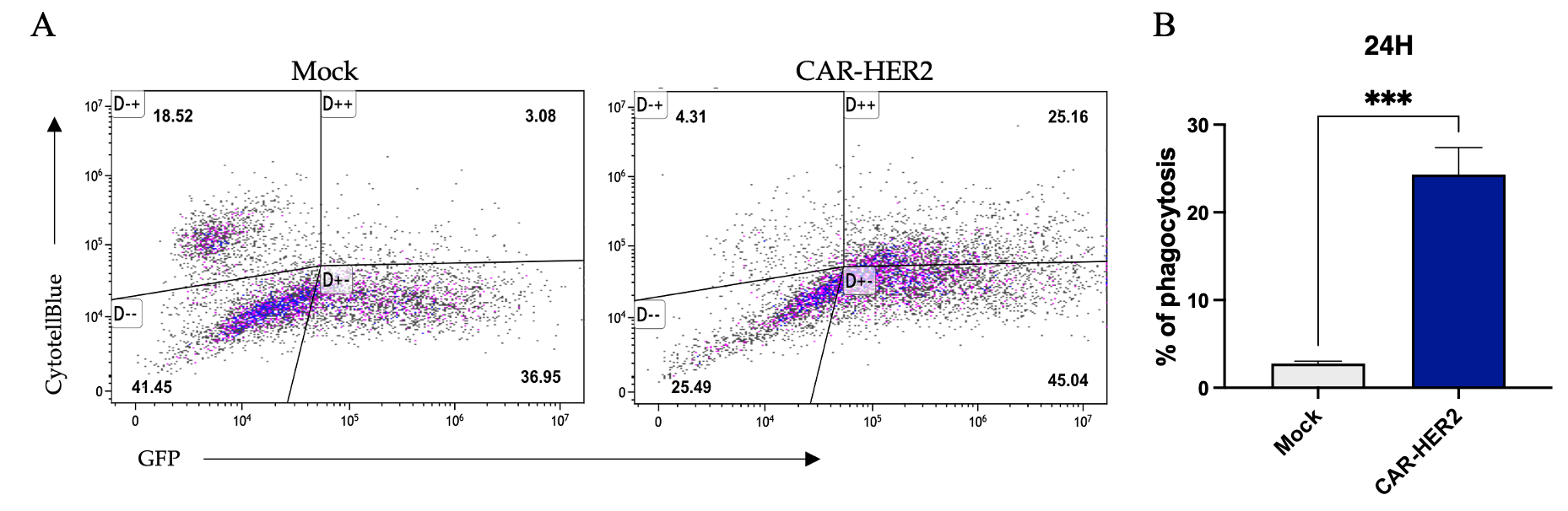


**Supp. Figure 10**: **CAR macrophages exhibit anti-tumor activity against SKBR3 HER2+ cancer cell line.** A) Representative FACS plots of phagocytosis after 24 hours of coculture with a 3:1 (macrophages/SKBR3 HER2+ cancer cells) ratio. The rate of phagocytosis is determined́ as the % of CytotellBlue+ and GFP+ cells among all cells. B) Quantification of phagocytosis of HER2+ cancer cells by mock and CAR-HER2 cells after 24 hours of coculture.


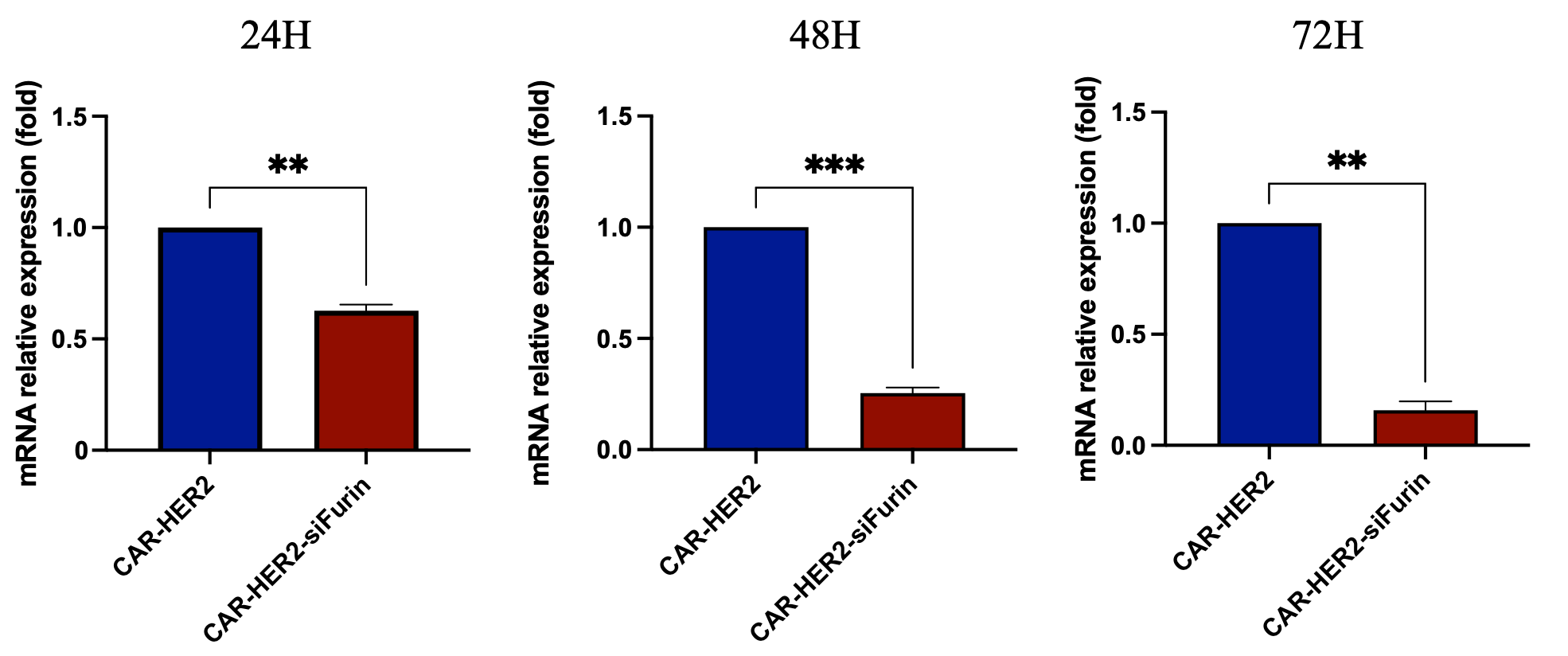


**Supp. Figure 11: Kinetic of furin inhibition in CAR-HER2 macrophages**. Relative mRNA levels of Furin after siRNA inhibition in CAR-HER2 macrophages. The fold change is expressed relative to the mRNA levels obtained from cells treated with the siRNA control (n=3).


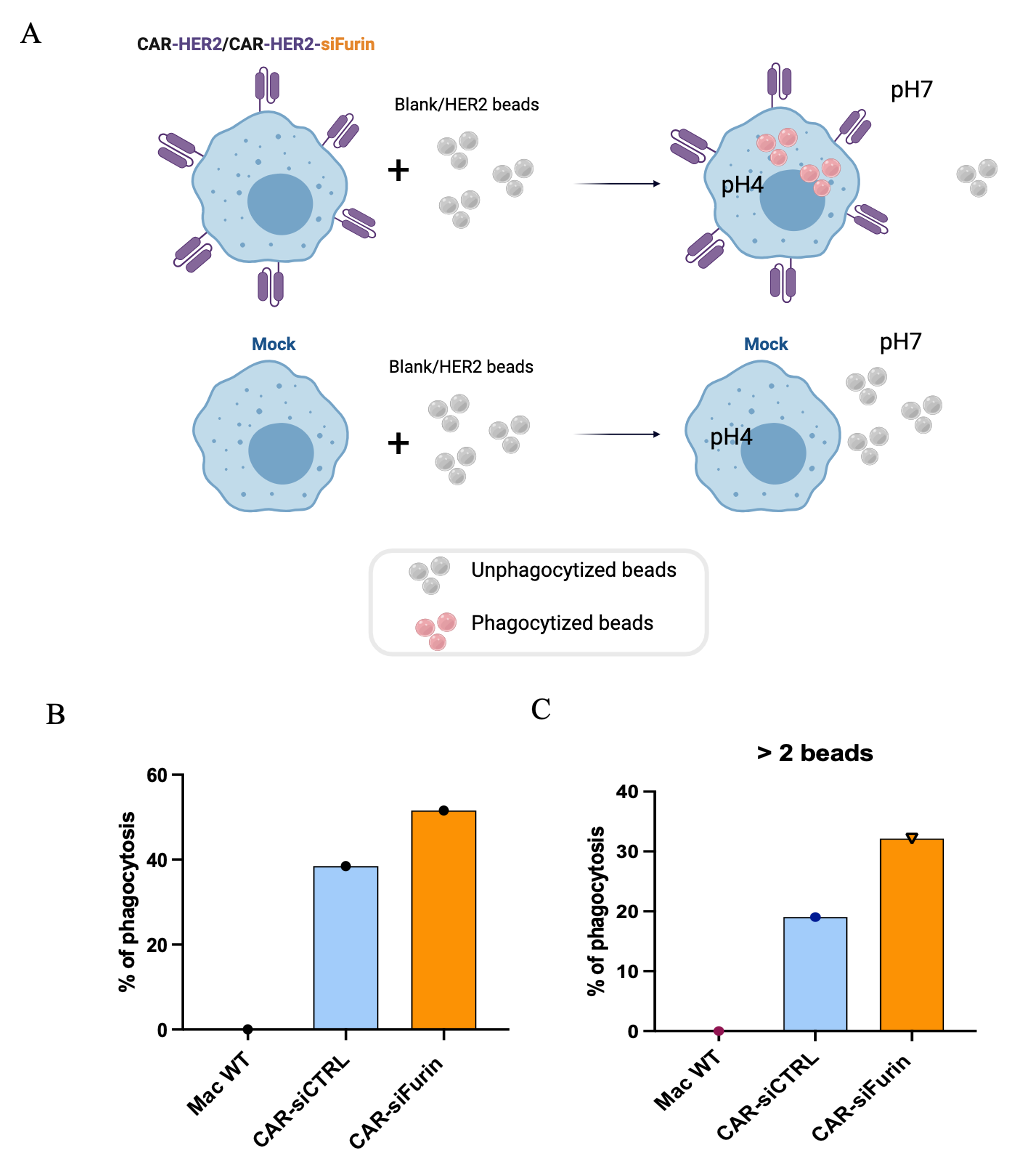


**Supp. Figure 12: Furin inhibition improves CAR-M phagocytic activity**. A) Phagocytosis bead assay concept. B) Quantification of internalization of HER2 beads and blank beads by WT macrophages, CAR-siControl or CAR-siFurin. 5 images were taken and quantified per condition (n=1). C) Quantification of the number of phagocytized beads per cell (1, 2 or more than 3 beads) in the 3 conditions (WT macrophages, CAR-siCtrl or CAR-siFurin).


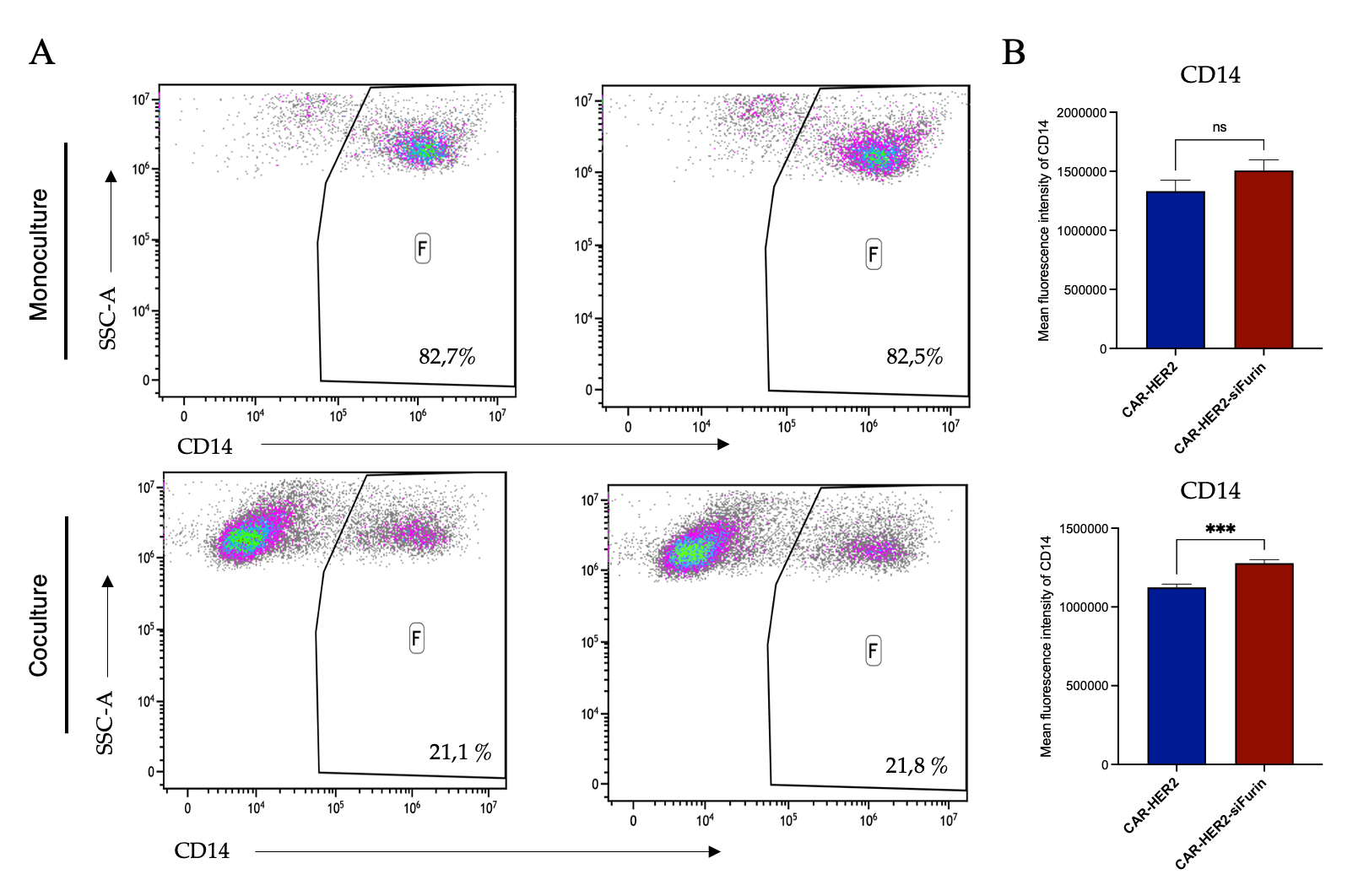


**Supp. Figure 13**: **CD14+ expression is increased in furin-inhibited CAR-M following coculture**. A) Scatter plots showing the frequency of CD14+ cells in CAR-HER2 and CAR-HER2-siFurin conditions, in both monoculture and coculture. B) The graphs show the mean fluorescence intensity (MFI) of CD14+ in CAR-HER2 and CAR-HER2-siFurin, in monoculture and coculture (n=3).


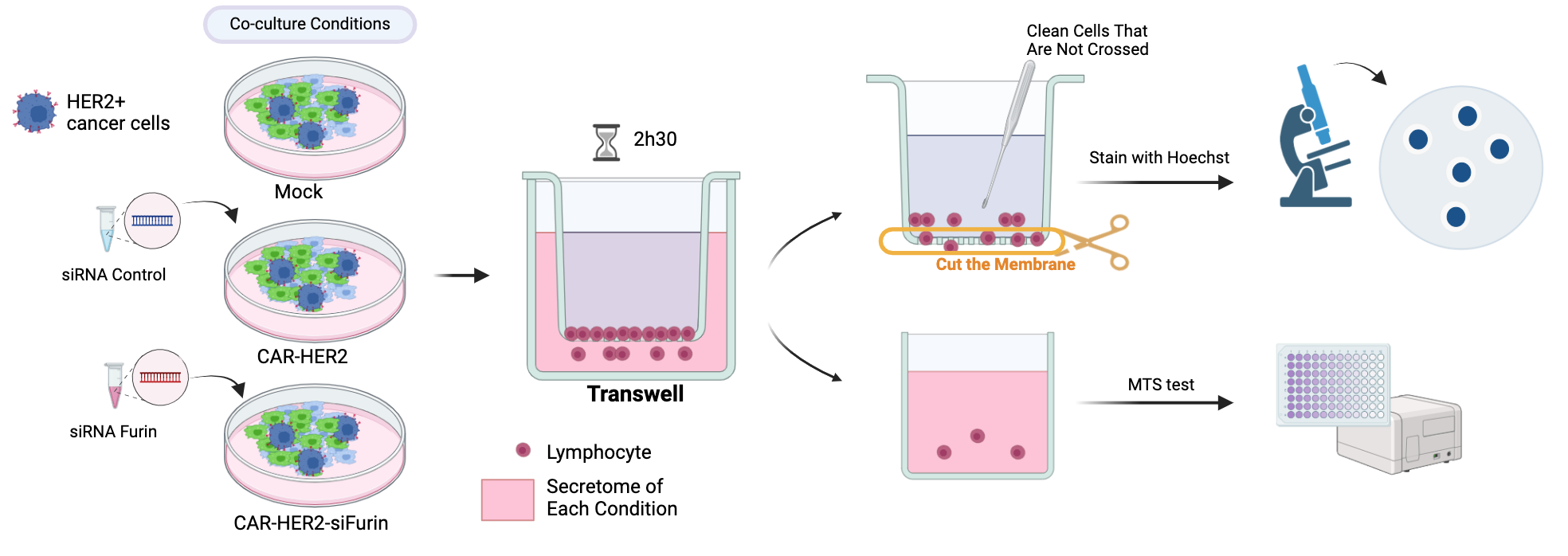


**Supp. Figure 14:**  **Description of the procedure for T-cell chemotaxis experiments**. After 24 hours of coculture between HER2+ cancer cells and mock, CAR or furin-inhibited CAR macrophages, the secretomes were collected, and T cells chemotaxis was assessed in the presence of the different secretomes. T-cell chemotaxis was evaluated using microscopy and MTS cell proliferation assay.


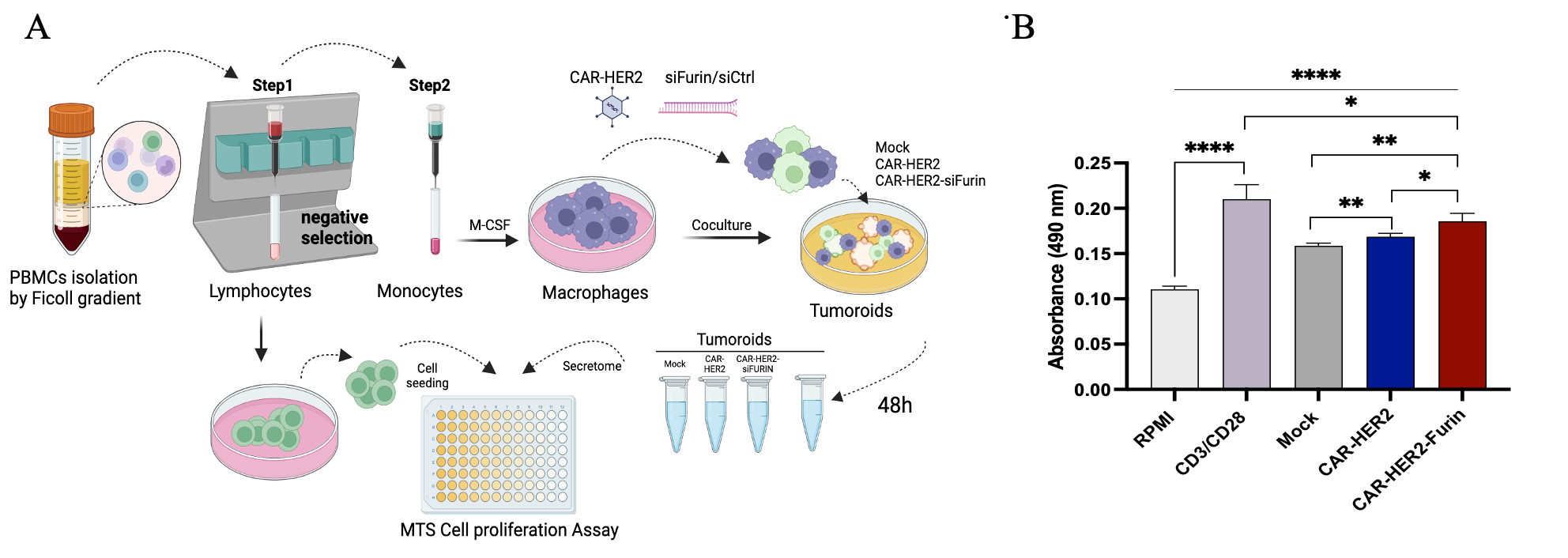


**Supp. Figure 15:** **Inhibition of Furin in CAR-Ms stimulates T lymphocytes.** A. Description of the procedure for purifying T cells from blood PBMCs and collecting the secretome from CAR-M/tumoroid cocultures. After 48 hours of coculture, the secretome was collected, and T cells were cultured in the presence of the secretome from tumoroids cocultured with mock, CAR-HER2 or CAR-HER2 siFurin macrophages. T-cell proliferation was measured using an MTS cell proliferation assay. B) Quantification of T lymphocyte proliferation under the indicated conditions. RPMI medium was used as a negative control, and CD3/CD28 beads were used as a positive control (n=3).

**
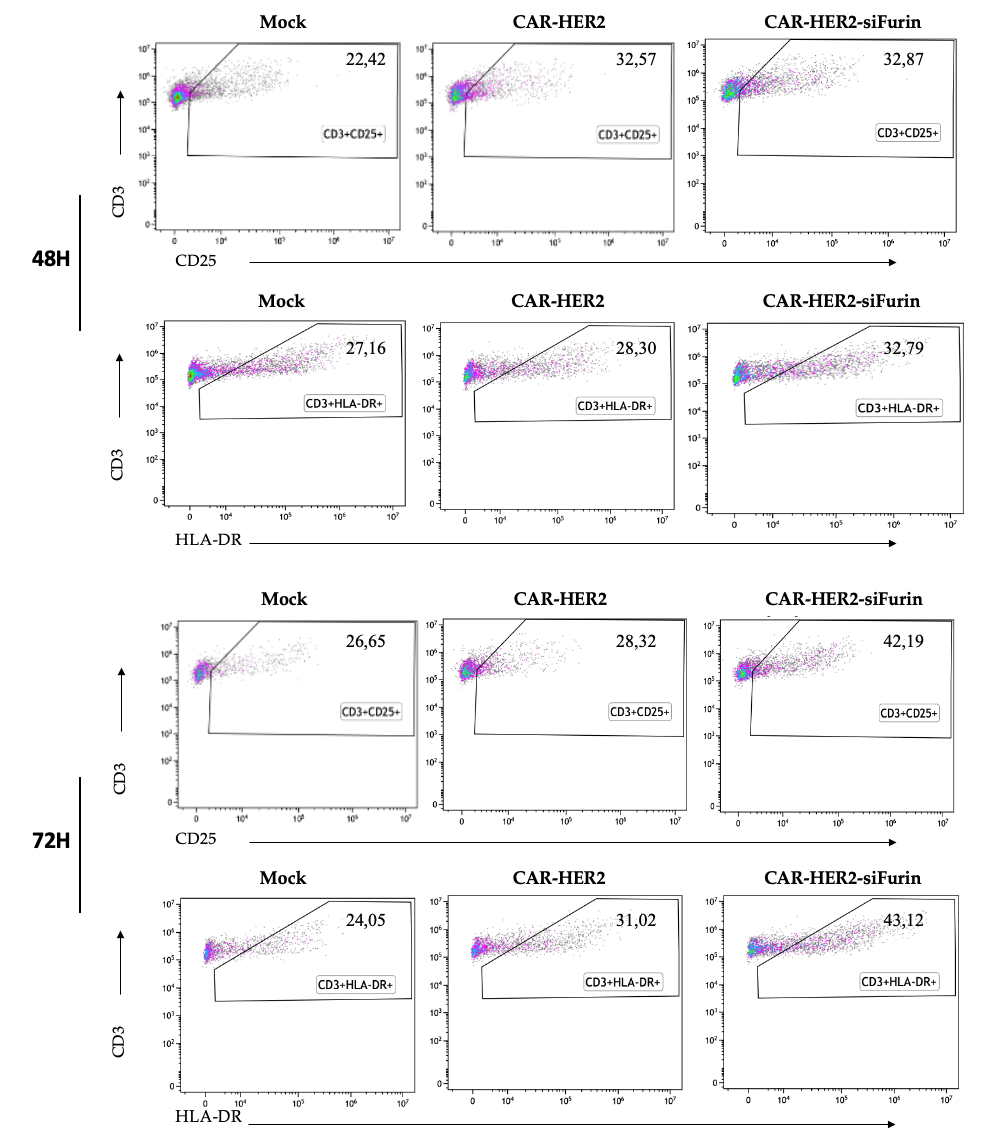
**

**Supp. Figure 16:** **Furin inhibition in CAR-Ms activates T cells**. Scatter plots illustrating the frequency of CD25+ and HLA-DR+ lymphocytes after co-culture with tumoroids and CAR-HER2, CAR-HER2-siFurin or Mock macrophages, after 24, 48, and 72h.


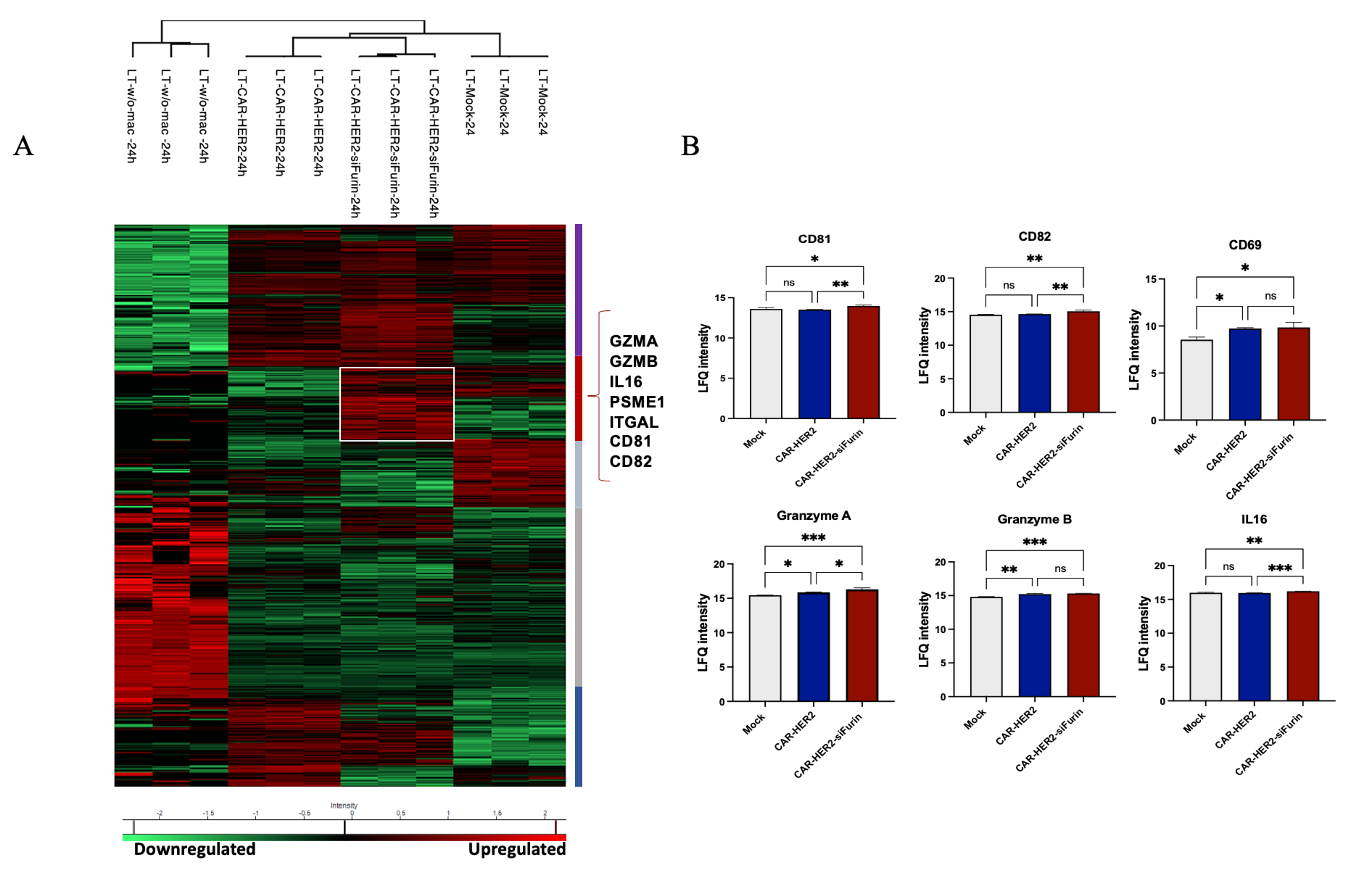


**Supp. Figure 17: Furin-inhibited CAR-M triggers increased expression of T cell activation markers.** A) The heatmap illustrates proteins that show significant differential expression across all conditions (LT with tumoroids, with and without mock, CAR or furin-inhibited CAR macrophages). B) Label-free quantification was used to identify activation markers in lymphocytes, with identification conducted at a 0.01 FDR threshold.

**Legends of supplementary tables**:

**Supp. table 1**: Total matrix extracted from the Perseus file containing the list of proteins identified in THP-1 macrophages after siRNA-mediated inhibition of furin and PC1/3.

**Supp. table 2**: List of overexpressed proteins in Clusters 1, 2, 3, 4 and 5 from the heatmap shown in Figure 2.

**Supp. table 3**: List of exclusive proteins expressed in THP-1 macrophages after siRNA-mediated inhibition of furin and PC1/3 (with reference to Figure 3).

**Supp. table 4:** Total matrix extracted from the Perseus file containing the list of proteins identified in CAR-Ms after siRNA-mediated inhibition of furin.

**Supp. table 5:** List of overexpressed proteins in clusters 1 and 2 from the heatmap shown in Figure 7.

**Supp. table 6**: Total matrix extracted from the Perseus file containing the list of proteins identified in T lymphocytes following co-culture with CAR-M-tumoroids.

**Supp. table 7**: List of proteins overexpressed in the cluster highlighted in Supp. Fig. 17.
