# Supplementary figures and images for: Inhibition of furin in CAR macrophages directs them toward a proinflammatory phenotype and enhances their antitumor activities
Source: Cell Death Dis. 2024 Dec 4;15(12):879. doi: 10.1038/s41419-024-07267-4 (PMC11618602; doi:10.1038/s41419-024-07267-4)

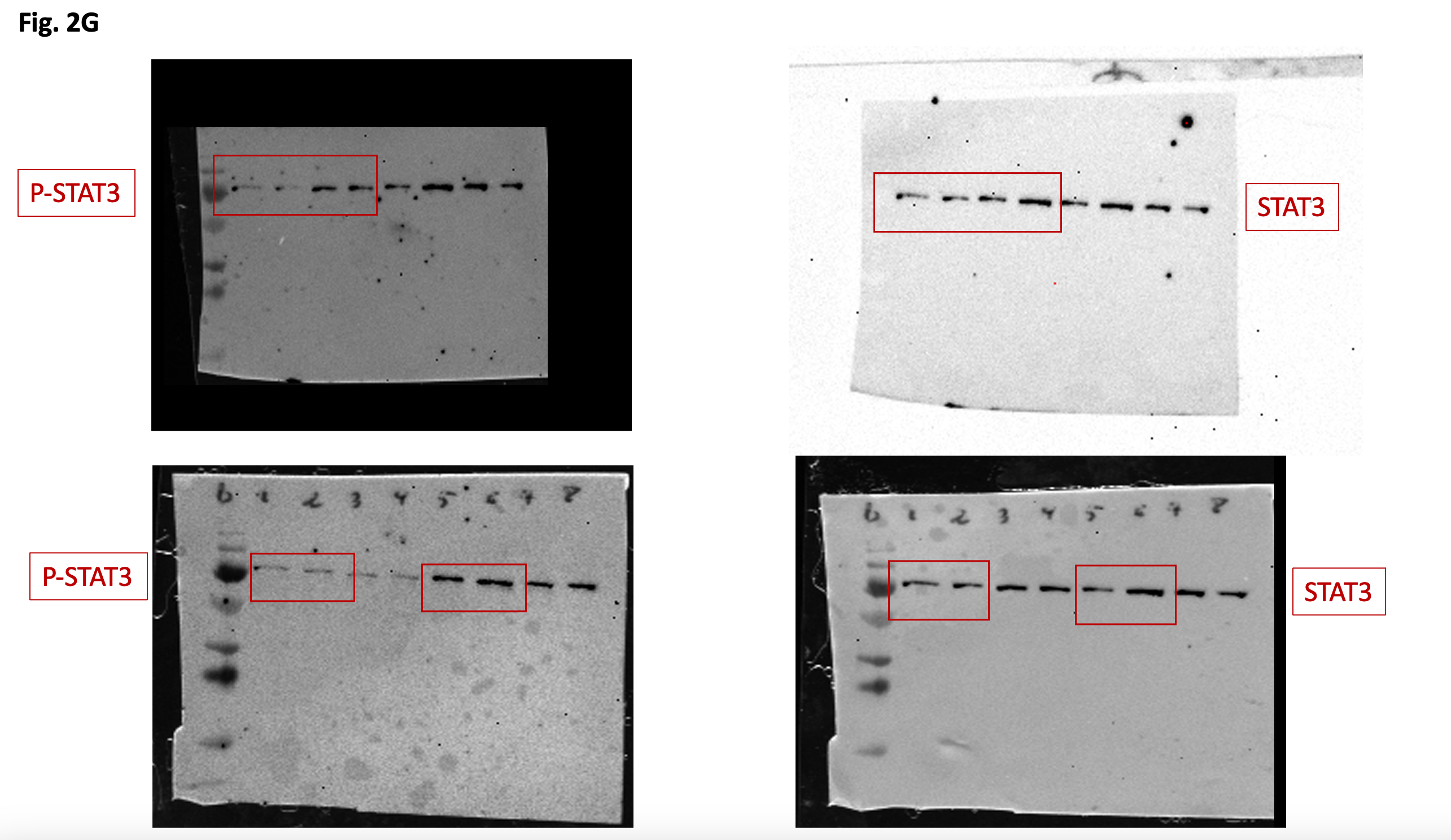


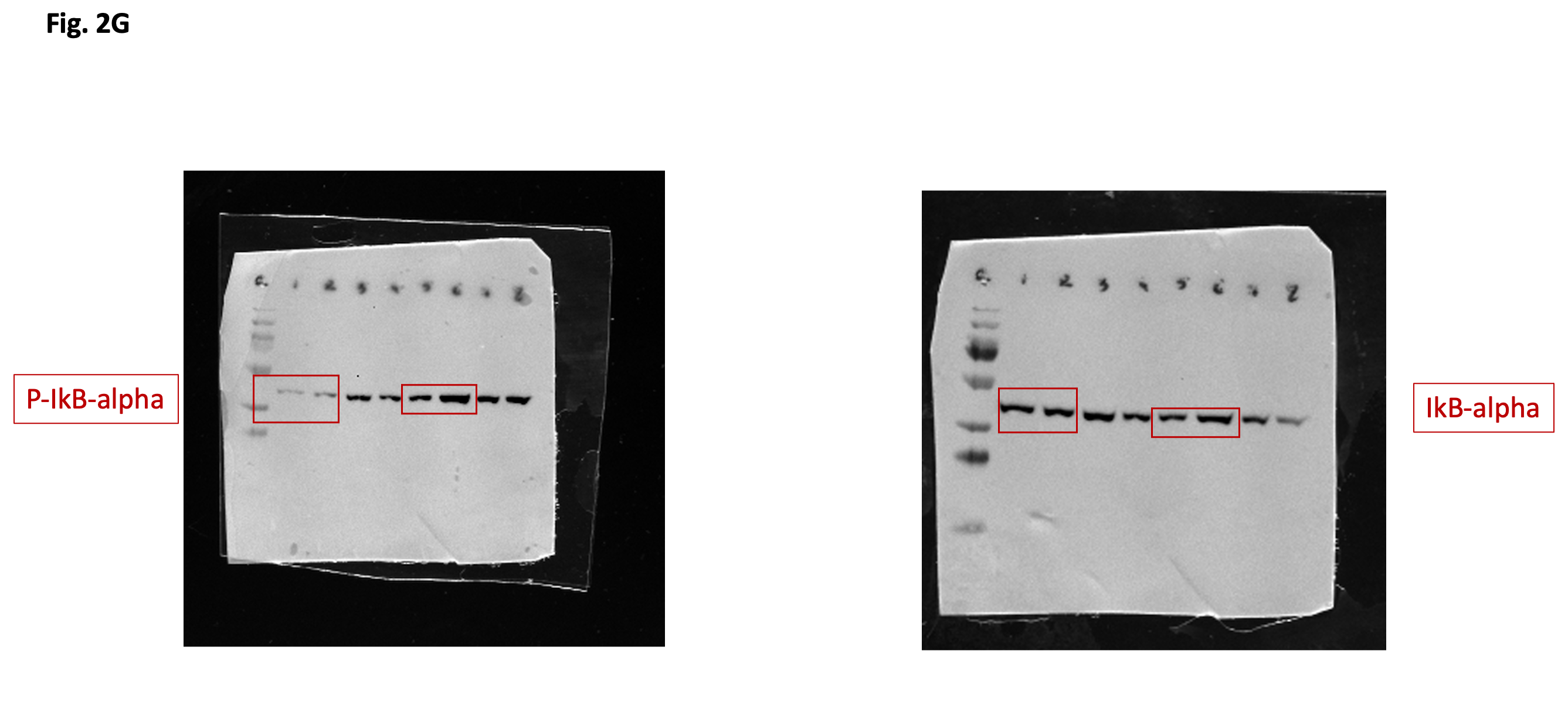


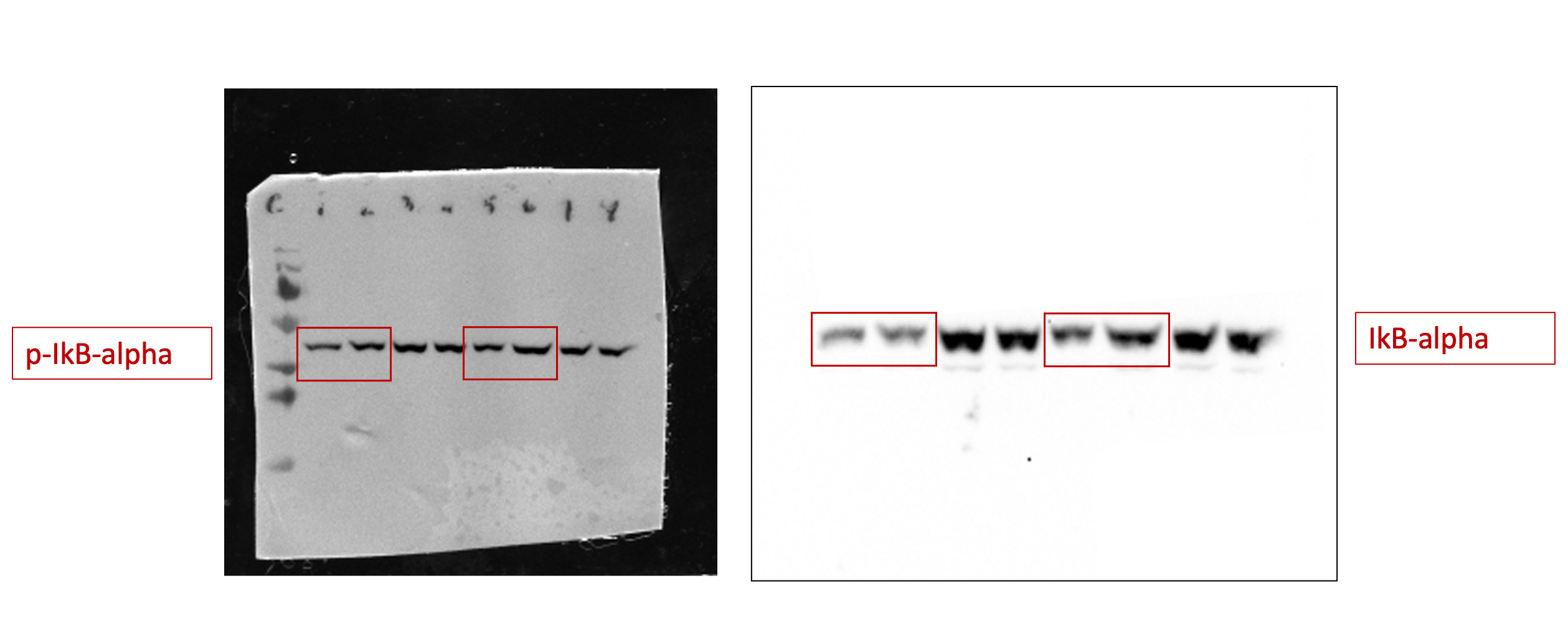

Supplement: Supplementary file 2 — Original data [file 41419_2024_7267_MOESM2_ESM.docx]
